# High-intensity interval training remodels perineuronal nets in the medial prefrontal cortex to drive microglial polarization and alleviate osteoarthritis pain

**DOI:** 10.1038/s41598-026-40823-w

**Published:** 2026-02-20

**Authors:** Changsheng Lin, Xiao Zhang, Ziqi Ye, Fang Zhou, Kaizong Huang, Shiting Zhu, Anliang Chen, Xueping Li

**Affiliations:** 1 Department of Rehabilitation Medicine, Nanjing First Hospital, Nanjing Medical University, Nanjing, China; 2 Department of Rehabilitation Medicine, Lianyungang Clinical College of Nanjing Medical University, Lianyungang, China; 3 Department of Clinical Pharmacology Lab, Nanjing First Hospital, Nanjing Medical University, Nanjing, China; 4 Department of Rehabilitation Medicine, Lianshui County People’s Hospital, Huaian, China

**Keywords:** Knee osteoarthritis, High-intensity interval training, Perineuronal nets, Microglial polarization, Neuroinflammation, Diseases, Immunology, Neurology, Neuroscience

## Abstract

Knee osteoarthritis (OA) is a leading cause of chronic pain and disability, yet the mechanisms linking central neuroinflammation to pain persistence remain poorly defined. This study identifies a novel perineuronal net (PNN)–microglia axis within the medial prefrontal cortex (mPFC) as a critical regulator of OA pain sensitization. Using a rat OA model, we demonstrate that high-intensity interval training (HIIT) exerts robust analgesic and disease-modifying effects, improving gait, reducing pain, and preserving cartilage integrity. HIIT markedly decreased PNN accumulation in the mPFC, driving microglial polarization away from a pro-inflammatory iNOS⁺ phenotype toward an anti-inflammatory Arg1⁺ phenotype, thereby mitigating central neuroinflammation. Importantly, pharmacological and enzymatic interventions confirmed that PNN remodeling precedes microglial phenotype switching, establishing a causal hierarchy in central inflammatory reprogramming. Parallel reductions in pro-inflammatory cytokines (IL-1β, TNF-α) and increases in IL-10 in both serum and synovial fluid underscore a systemic anti-inflammatory effect. Together, these findings reveal a previously unrecognized mechanism whereby exercise alleviates chronic pain by targeting central neuroimmune interactions, positioning HIIT as a promising non-pharmacological strategy for OA pain management through PNN-driven microglial modulation.

**Supplementary Information:**

The online version contains supplementary material available at 10.1038/s41598-026-40823-w.

## Introduction

Knee osteoarthritis (OA) is a prevalent degenerative joint disorder marked by ongoing pain, stiffness, and disability, representing a major global health burden^1^. Pain management remains a clinical challenge because symptoms arise from both peripheral joint inflammation and central sensitization^2,3^. Emerging evidence highlights the importance of central nervous system (CNS) changes, particularly neuroinflammation and maladaptive plasticity in the medial prefrontal cortex (mPFC), in sustaining chronic OA pain^4–6^.

Microglia, the primary immune cells in the CNS, are central to neuroinflammation and pain regulation^7–9^. They have the ability to differentiate into pro-inflammatory (M1) or anti-inflammatory (M2) phenotypes in response to environmental signals^10–12^. M1 microglia release cytokines such as interleukin-1β (IL-1β) and tumor necrosis factor-α (TNF-α), which sustain inflammation and pain sensitization, whereas M2 microglia release interleukin-10 (IL-10) to promote repair and resolution^11,13–15^. Skewed polarization toward M1 is closely associated with persistent pain, suggesting that restoring M2 dominance may provide analgesic benefits.

Perineuronal nets (PNNs), distinct extracellular matrix structures encircling inhibitory interneurons, maintain synaptic stability and limit plasticity^16–18^. Excessive PNN accumulation restricts adaptive remodeling and has been linked to enhanced microglial activation and pro-inflammatory bias^19,20^. However, whether PNN remodeling directly regulates microglial polarization in OA-related pain remains unclear.

Exercise, especially high-intensity interval training (HIIT), has shown efficacy in reducing OA pain and inflammation while improving joint function^21,22^. Both clinical and preclinical data suggest that HIIT exerts **s**ystemic and central anti-inflammatory effects, yet the underlying mechanisms remain incompletely defined^4,23^. In particular, the interaction between HIIT, PNN remodeling, and microglial polarization in the mPFC has not been systematically investigated.

This study addresses this gap by testing whether HIIT alleviates OA pain through suppression of PNN accumulation in the mPFC and subsequent reprogramming of microglia from a pro-inflammatory to an anti-inflammatory phenotype. Moreover, we investigate the causal sequence between PNN remodeling and microglial polarization. By integrating behavioral, histological, molecular, and pharmacological/enzymatic approaches, we propose a novel PNN–microglia axis underlying exercise-induced analgesia. These findings may establish a mechanistic basis for HIIT as a non-pharmacological strategy targeting central neuroimmune dysregulation in OA.

## Materials and methods

### Animals and OA model

Sprague-Dawley male rats aged 8 weeks were obtained from Qinglongshan Laboratory Animal Center (Nanjing, China). A total of 108 rats were used in this study. All animal experiments were approved by the Animal Care and Use Committee of Nanjing Medical University and conducted in accordance with institutional animal welfare guidelines. The rats were housed in cages with 3 animals per cage, in an environment with controlled temperature (22 ± 2 °C), humidity (55 ± 10%), and a 12-hour light/dark cycle. Environmental enrichment, including nesting material and chew toys, was provided. After one week of acclimatization, rats were anesthetized via intraperitoneal injection of 1.5% pentobarbital sodium (30 mg/kg). OA was induced by injecting 1 mg of monosodium iodoacetate (MIA; 20 mg/mL, dissolved in 50 µL sterile saline, Sigma-Aldrich, USA) into the right knee joint cavity. The control group received intra-articular injections of sterile saline (50 µL). On the day of the experiment, the animals weighed approximately 450 g. Animals were randomly assigned to different treatment groups, with each group consisting of 6 animals to ensure balanced distribution. The animals were divided into groups receiving ChABC treatment (via intracerebral microinjection) or minocycline treatment (via intraperitoneal injection), with further subdivisions based on the interventions (such as HIIT or control treatment). HIIT was performed starting 4 weeks after MIA injection, and it extended for a total of 6 weeks. Euthanasia and tissue collection were conducted 10 weeks post-MIA injection. The animals were anesthetized again via intraperitoneal injection of 1.5% pentobarbital sodium (30 mg/kg) and euthanized by cervical dislocation, and tissues from the right knee joint and the left mPFC were harvested for subsequent analysis. The experiments were conducted in multiple sessions, with animals being assigned to each session in a randomized and balanced manner. No animals were excluded from the trials. All methods were performed in accordance with the relevant guidelines and regulations.

### HIIT protocol

Rats in the HIIT groups were trained on a motorized small-animal treadmill (ZS-PT-5, Zhongshi Technology Co., Ltd., Beijing, China) five days per week (Monday to Friday) for a total of six weeks. Each session consisted of a 5-minute warm-up phase, during which the running speed was gradually increased from 5 to 15 m/min. This was followed by alternating intervals of high- and moderate-intensity running, repeated for six cycles. Each cycle included 30 s of high-intensity running at 26 m/min, immediately followed by 90 s of moderate-intensity running at 15 m/min, yielding a 2-minute cycle. Training ended with a 3-minute cool-down at 5 m/min, and each HIIT session lasted 20 min in total.

### Behavioral assessments

All behavioral assessments were performed by an investigator blinded to the experimental group allocation. Gait performance was evaluated using the CatWalk XT automated gait analysis system (Noldus Information Technology, Wageningen, Netherlands). Rats were allowed to traverse the walkway three times to obtain stable recordings, and parameters analyzed included body speed, stride length, step cycle, mean intensity difference between the left and right hindlimbs, paw print area difference, and duty cycle difference. Pain sensitivity was assessed using two methods. Mechanical allodynia was measured with an electronic von Frey aesthesiometer (IITC Life Science, Woodland Hills, CA, USA) by applying force to the plantar surface of the hind paw until withdrawal. Thermal hyperalgesia was assessed using a plantar test apparatus (Hargreaves method, Ugo Basile, Gemonio, Italy) to record paw withdrawal latency in response to radiant heat. All measurements were conducted in triplicate with 5-minute intervals, and the mean was used for further analysis. Knee joint swelling was evaluated by measuring the medio-lateral diameter of both knees with a digital caliper (Mitutoyo, Kawasaki, Japan) and calculating the difference between the right and left knees.

## Histological analysis

Knee joints were fixed in 4% paraformaldehyde for 48 h, decalcified in 10% EDTA (pH 7.4, Servicebio, G1105-500ML) for 4 weeks, dehydrated, cleared, and embedded in paraffin. Paraffin-embedded rat knee joint cartilage sections were prepared with a rotary microtome (Leica RM2235, Leica Microsystems, Germany) at 5 μm thickness. Staining procedures included Safranin O-Fast Green (Servicebio, G1053-100ML), HE (Servicebio, G1005-100ML), and Toluidine Blue (Servicebio, G1032-100ML). Cartilage degeneration was evaluated independently by two blinded observers using the OARSI and Modified Mankin scoring systems.

### Immunohistochemistry and immunofluorescence staining (IHC/IF)

For immunohistochemistry, paraffin-embedded rat knee joint cartilage sections were deparaffinized, rehydrated, and subjected to antigen retrieval using citrate buffer (pH 6.0, Servicebio, G1202) at 95 °C for 15 min. Endogenous peroxidase activity was blocked with 3% hydrogen peroxide for 10 min, followed by blocking with 5% bovine serum albumin (BSA) (Servicebio, G5001) for 30 min. Sections were incubated overnight at 4 °C with primary antibodies against COL2A1 (1:200, Santa Cruz Biotechnology, sc-52658) and MMP13 (1:200, Proteintech, 18165-1-AP). After PBS-T washes, sections were incubated with HRP-conjugated secondary antibodies (Proteintech, SA-00001-1/2) for 1 h at room temperature, developed using a DAB Substrate Kit (Servicebio, G1313-100T), and counterstained with hematoxylin. For immunofluorescence, 30 μm coronal sections from the mPFC were blocked in 5% BSA containing 0.3% Triton X-100 for 1 h at room temperature, then incubated overnight at 4 °C with combinations of Wisteria floribunda agglutinin (WFA, Sigma-Aldrich, L1516, 1:500), anti-Iba1 (1:200, Proteintech, 10904-1-AP), and anti-iNOS (1:200, Santa Cruz Biotechnology, sc-7271) or anti-Arg1 (1:200, Santa Cruz Biotechnology, sc-47715). Sections were washed and incubated with CoraLite647 Goat Anti-Mouse IgG (Proteintech, SA00014-10) and CoraLite594 Goat Anti-Rabbit IgG (Proteintech, SA00013-4), counterstained with antifade mounting medium containing DAPI (Servicebio, G1407-25ML), and imaged with a confocal microscope (Leica TCS SP8, Leica Microsystems, Germany).

### Western blotting

Tissues from the mPFC and knee cartilage were homogenized in RIPA lysis buffer (Boster, Wuhan, China, AR0102) containing protease inhibitor cocktail (Roche, Basel, Switzerland, 04693159001) on ice for 30 min, followed by centrifugation at 12,000 rpm for 15 min at 4℃. Protein concentrations were determined by the Bradford assay (Beyotime, Shanghai, China, P0006). Equal amounts of protein (30–50 µg) were separated on 10–12% SDS-PAGE gels (FuturePAGE, ACE Biotechnology, ET15420LGel), transferred to PVDF membranes (Millipore, Billerica, MA, USA, IPVH00010), blocked with 5% skim milk (BD Biosciences, Franklin Lakes, NJ, USA) for 1 h, and incubated overnight at 4 °C with primary antibodies against β-actin (1:5000, Servicebio, GB15003-100), COL2A1 (1:1000, Santa Cruz Biotechnology, sc-52658), MMP13 (1:1000, Proteintech, 18165-1-AP), iNOS (1:1000, Santa Cruz Biotechnology, sc-7271), Arg1 (1:1000, Santa Cruz Biotechnology, sc-47715), IL-10 (1:1000, Santa Cruz Biotechnology, sc-32815), IL-1β (1:1000, Santa Cruz Biotechnology, sc-12742), and TNF-α (1:1000, Santa Cruz Biotechnology, sc-52746). After TBST washes, membranes were incubated with HRP-conjugated secondary antibodies (1:5000, Proteintech, SA-00001-1/2) for 1 h, and protein bands were visualized using ECL luminescence reagent (Meilunbio, Dalian, China, MA0186-2) and imaged with a Bio-Rad ChemiDoc MP Imaging System (Bio-Rad, Hercules, CA, USA). Bands were quantified by ImageJ and normalized to β-actin.

### Elisa

Concentrations of IL-1β, TNF-α, and IL-10 in serum and synovial fluid were measured using ELISA kits (Servicebio, GER0002-48T, GER0004-48T, GER0003-48T) according to the manufacturer’s instructions. Under anesthesia, 50 µL of sterile saline was injected into the right knee joint cavity using a sterile 27-gauge needle, followed by the aspiration of 50 µL of synovial fluid. The individuals from which joint fluid was collected were the same as those used for tissue staining. Optical density was measured at 450 nm using a microplate reader (BioTek Instruments, Winooski, VT, USA).

### ChABC intracerebral microinjection into the mPFC

To achieve sustained degradation of PNNs during the six-week HIIT period, rats were anesthetized with isoflurane and placed in a stereotaxic apparatus. After a midline scalp incision and skull exposure, the left mPFC was targeted using the bregma as reference (AP + 3.0 mm, ML + 0.6 mm, DV -4.2/-4.0/-3.8 mm). A single track with three depths was used to enhance local diffusion. Small burr holes were drilled at the designated coordinates, and a microinjection needle was lowered to the target depth. ChABC solution (100 U/mL in PBS containing 0.1% BSA) or vehicle was infused at 0.6 µL per depth at a slow rate (approximately 0.1 µL/min) to minimize reflux. The needle was left in place for 5 min before withdrawal. Burr holes were sealed with bone wax, and the scalp was sutured. Animals recovered on a warming pad before being returned to their home cages. Injections were performed once weekly, one day before each HIIT session (i.e., on Sundays), for a total of six injections (weeks 1–6).

### Minocycline administration

To inhibit microglial activation during the experimental period, minocycline hydrochloride was freshly dissolved in sterile 0.9% saline at a concentration of 10–20 mg/mL, adjusted to pH 7.2–7.4, and filtered through a 0.22 μm membrane. The solution was administered by intraperitoneal injection at a dose of 50 mg/kg once per training day, 30 min prior to each HIIT session (5 days per week, Monday to Friday) for 6 consecutive weeks. In non-HIIT groups, injections were given at the same circadian time points and frequency as the HIIT groups. Control animals received an equal volume of vehicle. The final injection was given on the last training day of the HIIT protocol, and tissue assessments were conducted 24 h later to minimize acute drug effects.

### Statistical analysis

Statistical analyses were performed using GraphPad Prism 9.0 (GraphPad Software, San Diego, CA, USA). Data are presented as mean ± SD from at least three independent experiments. Comparisons between two groups were made using an unpaired two-tailed Student’s t-test, while one-way ANOVA followed by Bonferroni’s post hoc test was used for multiple group comparisons. Statistical significance was set at *p* < 0.05. Details on statistical methods and sample sizes are provided in the figure legends.

## Results

### HIIT improved gait performance, reduced pain sensitivity, and attenuated knee swelling in OA rats

**Fig. 1:**
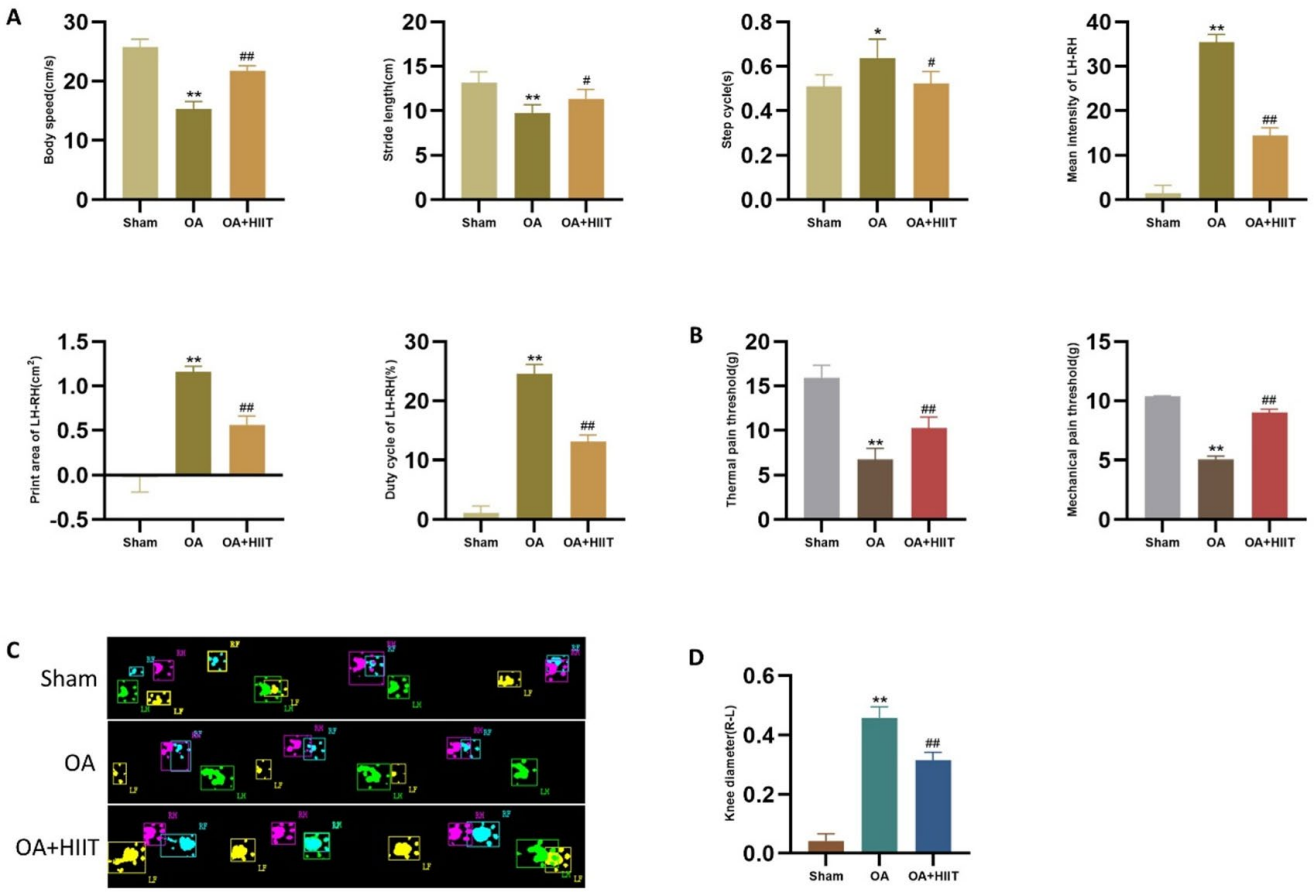
HIIT improved gait performance, reduced pain sensitivity, and attenuated knee swelling in OA rats. (**A**) Gait parameters, including body speed, stride length, step cycle, mean intensity of left minus right hindlimb, print area of left minus right hindlimb, and duty cycle of left minus right hindlimb (*n* = 6). (**B**) Thermal and mechanical pain sensitivity (*n* = 6). (**C**) Schematic diagram of rat gait analysis. (**D**) Knee diameter measurement (*n* = 6). Statistical methods used: One-way ANOVA. *p < 0.05 vs. the sham group, **p < 0.01 vs. the sham group, #*p* < 0.05 vs. the OA group, ##*p* < 0.01 vs. the OA group, Data are represented as mean ± SD.

Figure 1 shows the evaluation of gait characteristics, pain sensitivity, and knee joint swelling in rats. First, gait parameters-including body speed, stride length, step cycle, mean intensity difference (left minus right hindlimb), paw print area difference (left minus right hindlimb), and duty cycle difference (left minus right hindlimb)-were measured to assess functional performance (Fig. 1A). Rats in the OA group displayed significantly impaired gait parameters compared with the sham group, while these impairments were markedly alleviated after six weeks of HIIT. Additionally, thermal and mechanical pain sensitivity tests demonstrated increased pain sensitivity in the OA group relative to the sham group, and this heightened sensitivity was significantly improved by HIIT intervention (Fig. 1B). Representative schematic diagram of rat gait analysis is illustrated in Fig. 1C. Finally, knee diameter measurements showed significant knee joint swelling in OA rats compared to the sham group, with substantial reduction observed following HIIT (Fig. 1D).

### HIIT alleviated cartilage degeneration

**Fig. 2:**
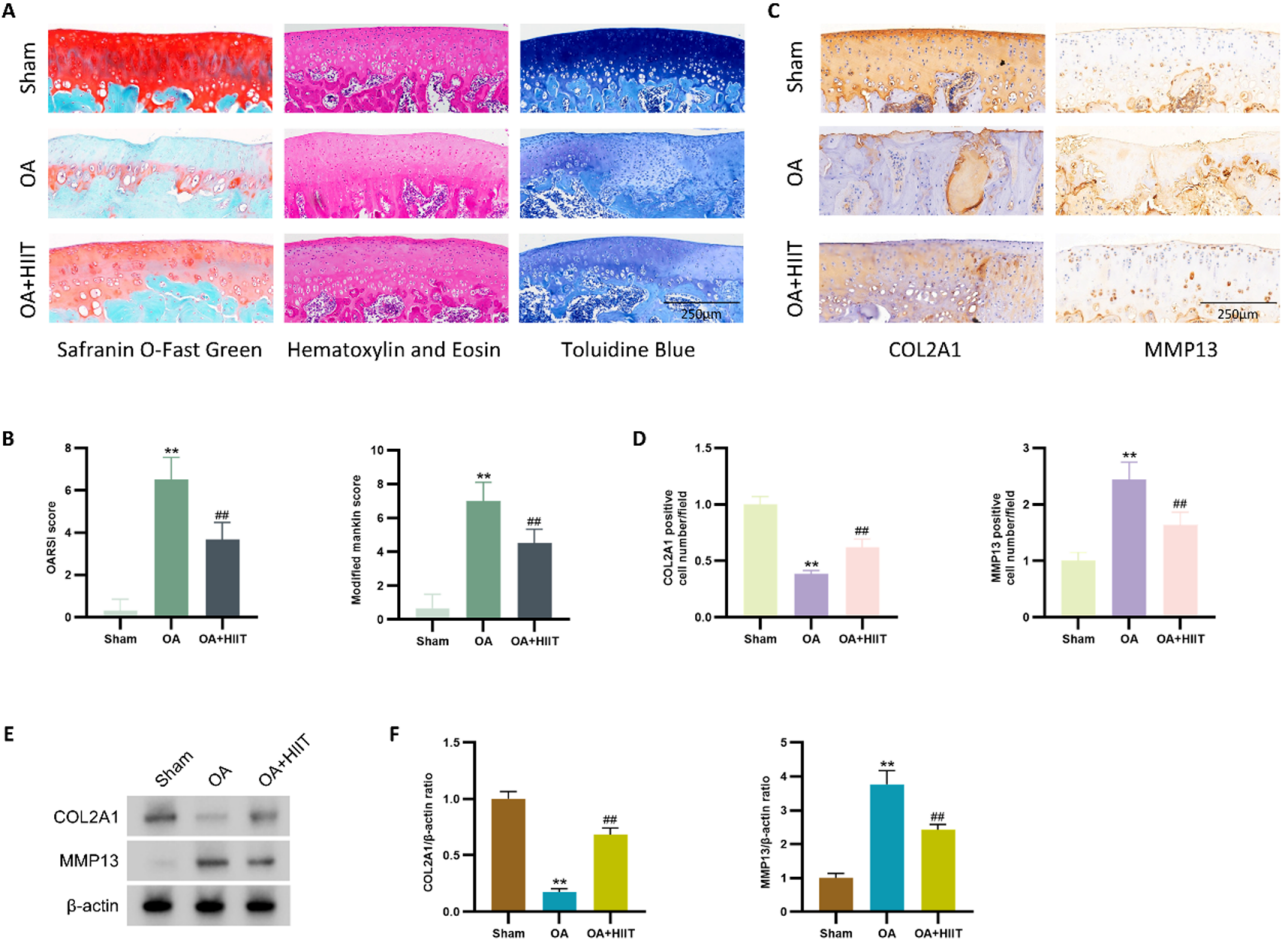
HIIT ameliorated cartilage degeneration. (**A** and **B**) Representative images of Safranin O-Fast Green staining, HE staining, and Toluidine Blue staining of knee cartilage (scale bar: 200 μm), along with quantitative analyses using OARSI and Modified Mankin scores (*n* = 6). (**C** and **D**) Immunohistochemistry staining and quantitative analysis of COL2A1 and MMP13 expression in knee cartilage (scale bar: 100 μm) (*n* = 6). (**E** and **F**) Western blot analysis and quantification of COL2A1 and MMP13 protein levels (*n* = 6). Statistical methods used: One-way ANOVA. **p < 0.01 vs. the sham group, ##*p* < 0.01 vs. the OA group, Data are represented as mean ± SD.

To further investigate whether HIIT could attenuate OA progression in vivo, monosodium iodoacetate (MIA) was injected into rat knee joints to establish an OA model^24^. Safranin O-Fast Green, Hematoxylin and Eosin (HE), and Toluidine Blue staining showed that MIA injection led to severe cartilage degeneration, while HIIT markedly alleviated these pathological changes (Fig. 2A and B). Furthermore, Osteoarthritis Research Society International (OARSI) and Modified Mankin scores quantitatively confirmed significant cartilage deterioration in the OA group relative to the sham group. In contrast, HIIT significantly lowered these scores, indicating reduced cartilage damage. Immunohistochemical staining demonstrated decreased expression of collagen type Ⅱ alpha 1 chain (COL2A1) and increased expression of matrix metalloproteinase-13 (MMP13) in the OA group relative to the sham group. HIIT intervention notably reversed these trends, increasing COL2A1 expression and reducing MMP13-positive staining cells (Fig. 2C and D).

Consistent with immunohistochemistry results, Western blot analysis showed decreased COL2A1 protein levels and increased MMP13 protein levels in the OA group. However, HIIT significantly elevated COL2A1 expression and reduced MMP13 levels (Fig. 2E and F). Collectively, these data suggest that HIIT effectively attenuated MIA-induced cartilage degeneration in rats.

### HIIT modulated inflammatory cytokine profiles

**Fig. 3:**
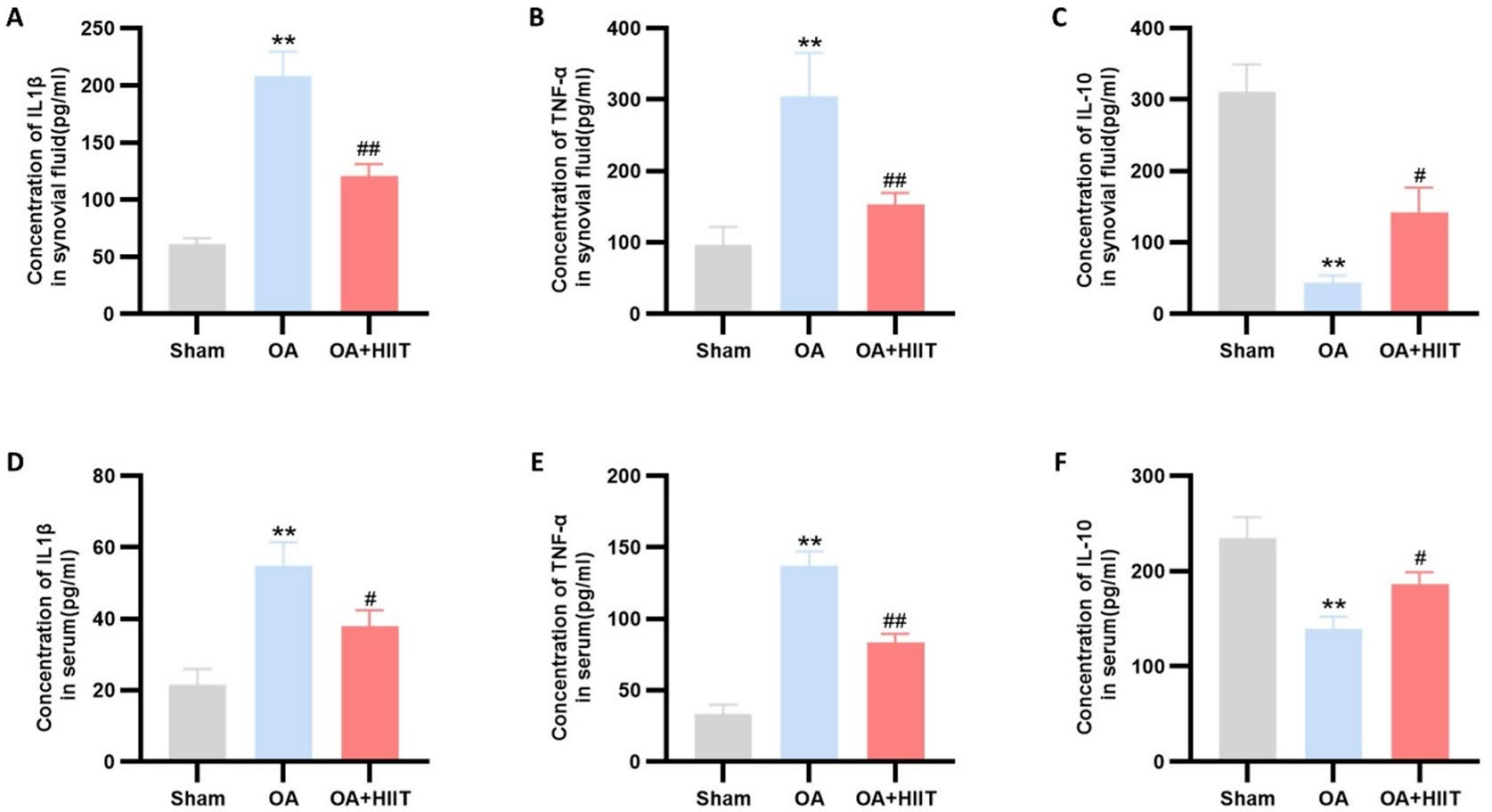
HIIT modulated inflammatory cytokine profiles. (**A**-**C**) IL-1β (**A**), TNF-α (**B**), and IL-10 (**C**) levels in rat synovial fluid (*n* = 6). (**D**-**F**) IL-1β (**D**), TNF-α (**E**), and IL-10 (**F**) levels in rat serum (*n* = 6). Statistical methods used: One-way ANOVA. **p < 0.01 vs. the sham group, #*p* < 0.05 vs. the OA group, ##*p* < 0.01 vs. the OA group. Data are represented as mean ± SD.

To examine the impact of HIIT on both systemic and joint inflammation in OA, we assessed the concentrations of key inflammatory cytokines in rat synovial fluid and serum. As shown in Fig. 3A and C, the OA group exhibited significantly elevated concentrations of pro-inflammatory cytokines IL-1β and TNF-α, along with attenuated levels of the anti-inflammatory cytokine IL-10 in synovial fluid, compared with the sham group. Comparable results were obtained in serum samples (Fig. 3D and F).

Following HIIT intervention, IL-1β and TNF-α levels were substantially decreased, while IL-10 levels were notably elevated in both synovial fluid and serum, suggesting an overall anti-inflammatory effect. These findings indicate that HIIT effectively alleviates the inflammatory response associated with OA.

### HIIT reduced PNN accumulation and promoted microglial M2 polarization in the mPFC

**Fig. 4:**
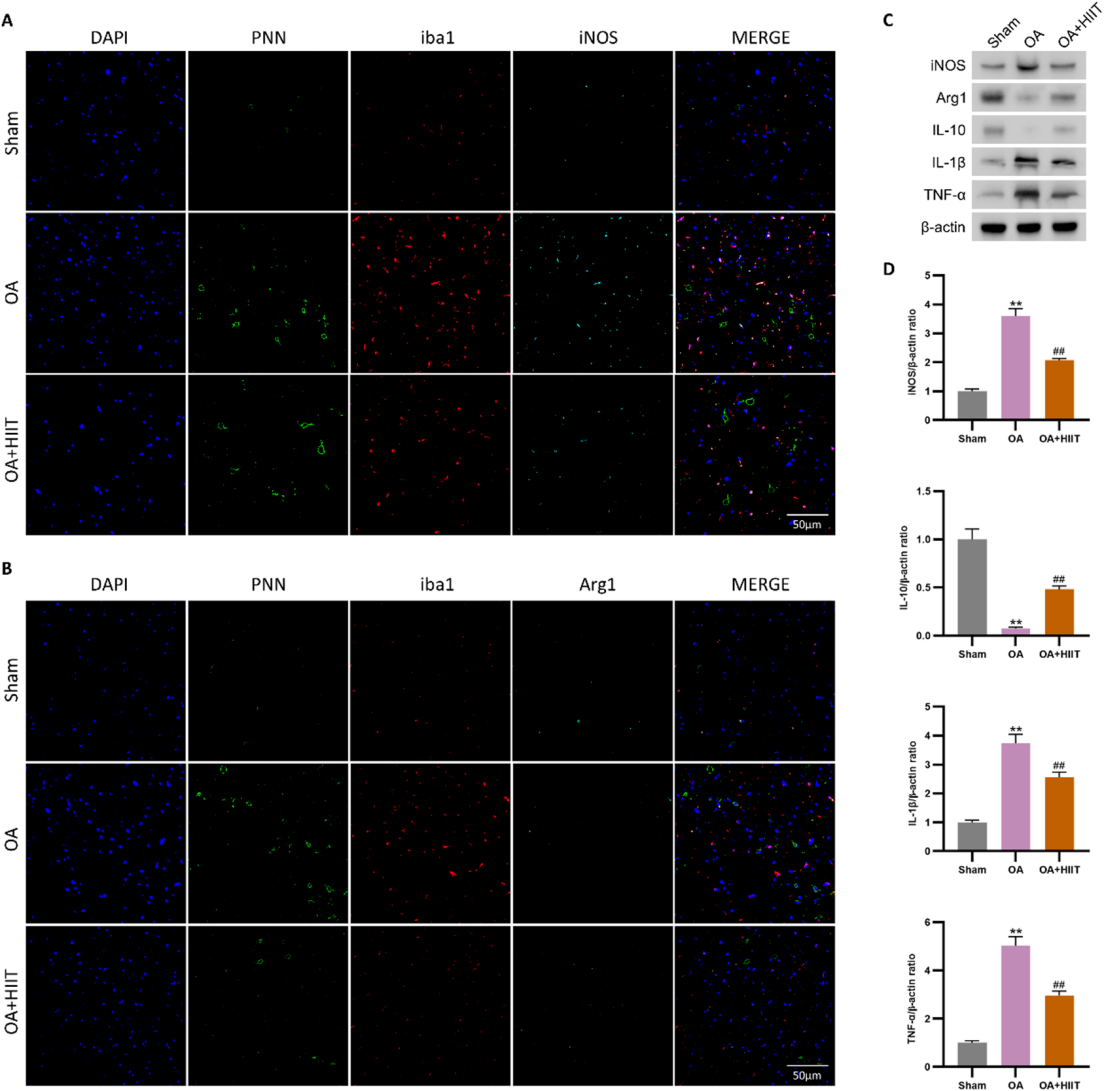
HIIT reduced PNN accumulation and promoted microglial M2 polarization in the mPFC. (A and B) Triple immunofluorescence staining in the mPFC showing PNN, Iba1, and iNOS (**A**) or PNN, Iba1, and Arg1 (**B**). OA rats showed increased PNN expression and pro-inflammatory (Iba1 + iNOS⁺) microglia, while HIIT reduced PNN levels and promoted anti-inflammatory (Iba1 + Arg1⁺) phenotypes. (C and D) Protein expression of iNOS, Arg1, IL-10, IL-1β, and TNF-α in the mPFC, detected by western blot (**C**) and quantified by bar graphs (**D**) (*n* = 6). Statistical methods used: One-way ANOVA. **p < 0.01 vs. the sham group, ##*p* < 0.01 vs. the OA group. Data are represented as mean ± SD.

To evaluate the regulatory effect of HIIT on the mPFC in osteoarthritic rats, we examined PNN expression, inflammatory cytokines, and microglial polarization. As shown in Fig. 4A and B, OA induced a marked increase in PNN expression along with enhanced colocalization of Iba1 with iNOS, indicating a pro-inflammatory microglial state^25^. Meanwhile, the expression of the anti-inflammatory marker Arg1 was suppressed^26^. HIIT intervention significantly attenuated PNN accumulation and reduced the proportion of Iba1 + iNOS⁺ cells, while enhancing Iba1 + Arg1⁺ microglia.

Western blot analysis of mPFC tissues (Fig. 4C and D) showed elevated iNOS, IL-1β, and TNF-α, and decreased IL-10 and Arg1 expression in OA rats compared to the sham group. These changes were reversed following HIIT. Collectively, these findings demonstrate that HIIT mitigates OA-induced neuroinflammation, downregulates aberrant PNN expression, and promotes a shift toward an anti-inflammatory microglial phenotype.

### PNNs degradation and HIIT alleviate neuroinflammation in the mPFC of OA rats

**Fig. 5:**
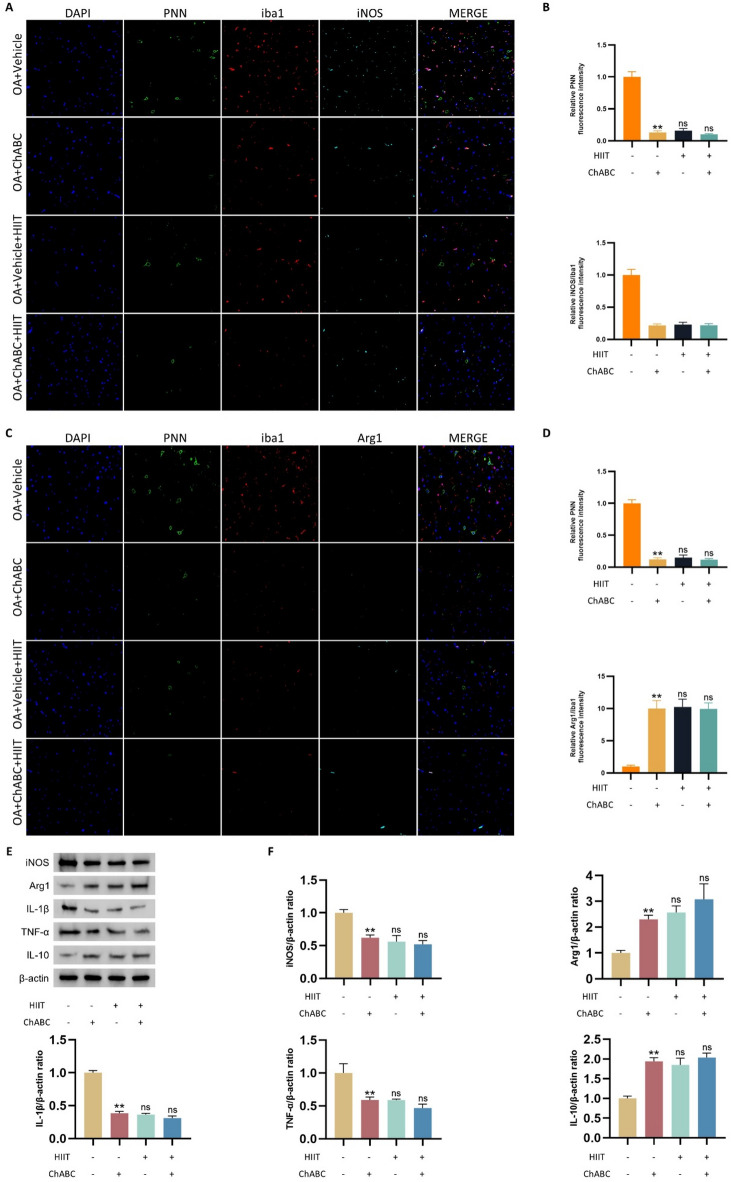
PNNs degradation and HIIT alleviate neuroinflammation in the mPFC of OA rats. (**A**-**D**) Representative triple immunofluorescence staining images of the mPFC, showing the distribution patterns of PNNs, Iba1 (microglial marker), and iNOS (**A**) or Arg1 (**C**). Quantification of panel A is shown in (**B**), and quantification of panel C is shown in (**D**) (*n* = 6). (**E** and **F**) Western blot analysis and corresponding quantification of inflammatory markers, including iNOS, Arg1, IL-1β, TNF-α, and IL-10 in mPFC tissue lysates (*n* = 6). Statistical methods used: One-way ANOVA. **p < 0.01 vs. the OA+Vehicle group, ns not significant vs. the OA+ChABC group.Data are presented as mean ± SD.

To explore the involvement of PNNs and HIIT in modulating central neuroinflammation associated with OA, rats underwent right knee OA modeling followed by microinjection of Chondroitinase ABC (ChABC) into the left mPFC to locally degrade PNNs. An additional group received systemic HIIT intervention, and another received the combined treatment. Triple immunofluorescence staining revealed that, compared to the OA+Vehicle group, PNN intensity was substantially decreased in the OA+ChABC group. This was accompanied by decreased Iba1-positive microglia and a shift in polarization: lower iNOS and higher Arg1 expression within Iba1 + cells (Fig. 5A and D). These findings indicate that local degradation of PNNs in the left mPFC attenuates microglial activation and promotes an anti-inflammatory phenotype.

Western blot analysis further confirmed these changes, showing reduced iNOS, IL-1β, and TNF-α expression and increased Arg1 and IL-10 levels in the OA+ChABC group (Fig. 5E and F). The HIIT group showed similar molecular changes, although direct comparison with OA+Vehicle was not performed in this figure. This interpretation was based on previous results demonstrating that HIIT effectively alleviates OA-related inflammation. No additive effect was observed in the combined ChABC+HIIT group compared to ChABC alone. Collectively, these results suggest that both local PNN degradation and systemic HIIT independently alleviate OA-related neuroinflammation in the mPFC, primarily by modulating microglial activation and promoting anti-inflammatory polarization.

### PNN degradation and HIIT alleviate OA-related joint dysfunction and inflammation

**Fig. 6:**
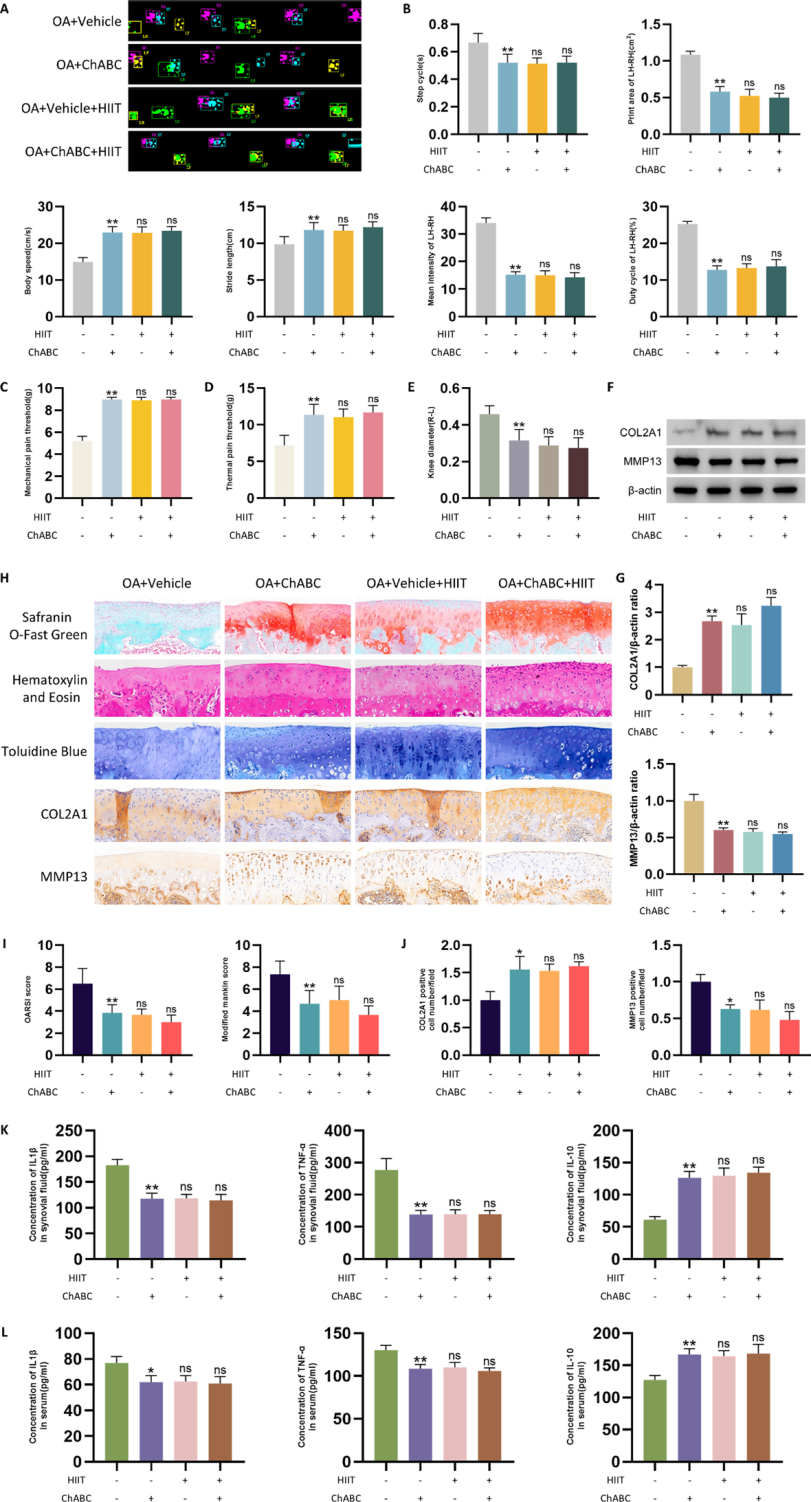
PNN degradation and HIIT alleviate OA-related joint dysfunction and inflammation. (**A**) Schematic diagram of rat gait analysis. (**B**) Gait parameters, including body speed, stride length, step cycle, mean intensity of left minus right hindlimb, print area of left minus right hindlimb, and duty cycle of left minus right hindlimb (*n* = 6). (**C** and **D**) Mechanical and thermal pain sensitivity (*n* = 6). (**E**) Knee diameter measurement (difference between right and left knees) (*n* = 6). (**F** and **G**) Western blot analysis and quantification of COL2A1 and MMP13 expression in knee cartilage (*n* = 6). (**H**) Representative images of Safranin O-Fast Green staining, HE staining, Toluidine Blue staining, and immunohistochemistry staining for COL2A1 and MMP13 in knee cartilage (scale bar: 200 μm for histology; 100 μm for IHC). (**I** and **J**) Quantitative analyses using OARSI and Modified Mankin scores (**I**), and immunohistochemistry analysis of COL2A1 and MMP13 (**J**) (*n* = 6). (**K** and **L**) ELISA analysis of IL-1β, TNF-α, and IL-10 levels in synovial fluid (**K**) and serum (**L**) (*n* = 6). Statistical methods used: One-way ANOVA. *p < 0.05, **p < 0.01 vs. the OA+Vehicle group; ns: not significant vs. the OA+ChABC group. Data are represented as mean ± SD.

To determine whether PNN degradation and HIIT improve joint function and peripheral inflammation in OA rats, gait performance, pain sensitivity, joint swelling, cartilage integrity, and cytokine levels were assessed across groups. Compared to the OA+Vehicle group, the OA+ChABC group exhibited improved gait parameters, including increased body speed and stride length, reduced step cycle, and decreased.

asymmetry between left and right hindlimbs in intensity, print area, and duty cycle (Fig. 6A and B). Mechanical and thermal pain sensitivity were also ameliorated in the OA+ChABC group (Fig. 6C and D).

Knee diameter measurement showed reduced swelling in the OA+ChABC group compared to OA+Vehicle (Fig. 6E). Western blot analysis and quantification revealed increased COL2A1 and decreased MMP13 expression in knee cartilage (Fig. 6F and G). Histological staining, including Safranin O-Fast Green, HE, and Toluidine Blue, demonstrated improved cartilage structure^27^, and immunohistochemistry confirmed higher COL2A1 and lower MMP13 levels (Fig. 6H). These findings were supported by lower OARSI and Modified Mankin scores and quantification of immunohistochemistry (Fig. 6I and J). Additionally, ELISA measurements of IL-1β, TNF-α, and IL-10 in synovial fluid (Fig. 6K) and serum (Fig. 6L) showed trends consistent with the previous results, where the OA+ChABC group exhibited lower IL-1β and TNF-α levels and higher IL-10 levels, indicating a reduction in peripheral inflammation in the ChABC-treated group.

No significant improvements in gait, pain, swelling, cartilage markers, and cytokine levels were observed in the OA+Vehicle+HIIT and OA+ChABC+HIIT groups compared to the OA+ChABC group. These findings suggest that combining HIIT with ChABC treatment did not result in additional benefits over ChABC treatment alone.

### PNN remodeling precedes microglial polarization

**Fig. 7:**
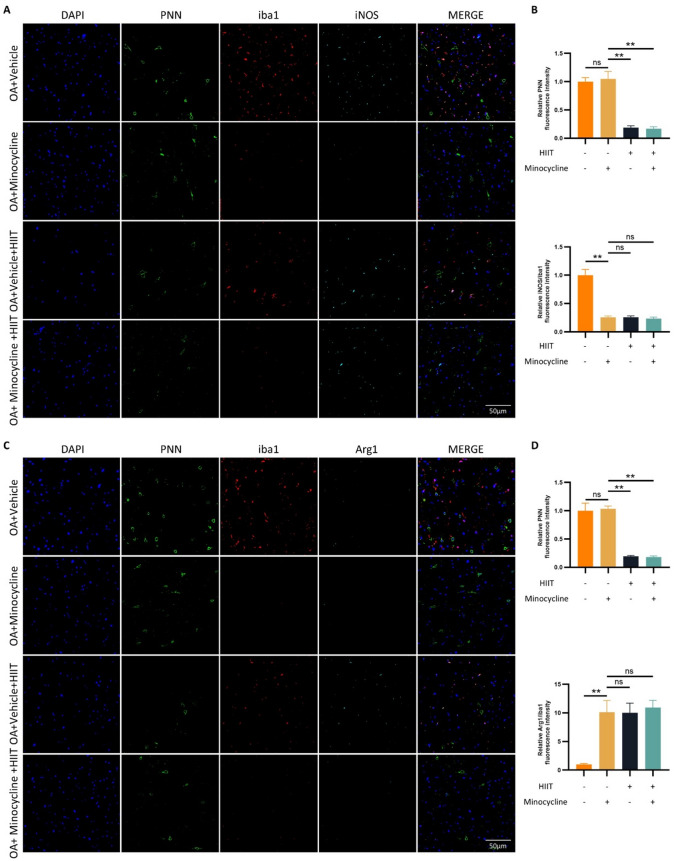
PNN remodeling precedes microglial polarization. (**A**-**D**) Representative triple immunofluorescence staining images of the mPFC, showing PNN, Iba1, and iNOS (**A**), or PNN, Iba1, and Arg1 (**C**). Quantification of panel A is shown in (**B**), and quantification of panel C is shown in (**D**) (*n* = 6). Statistical methods used: One-way ANOVA. **p < 0.01. Data are presented as mean ± SD.

To further clarify the causal relationship between PNN remodeling and microglial polarization, Minocycline was used to pharmacologically inhibit microglial activation. Triple immunofluorescence staining of the mPFC revealed that Minocycline treatment suppressed microglial activation, with no change in PNN expression in the OA+Minocycline group (Fig. 7A and D). In contrast, PNN reduction was observed in both HIIT-treated groups, regardless of Minocycline co-administration. These findings suggest that HIIT does not act through microglial inhibition to alter PNN structure, but rather, PNN remodeling likely precedes and mediates HIIT-induced microglial phenotype switching.

### Proposed mechanism

**Fig. 8:**
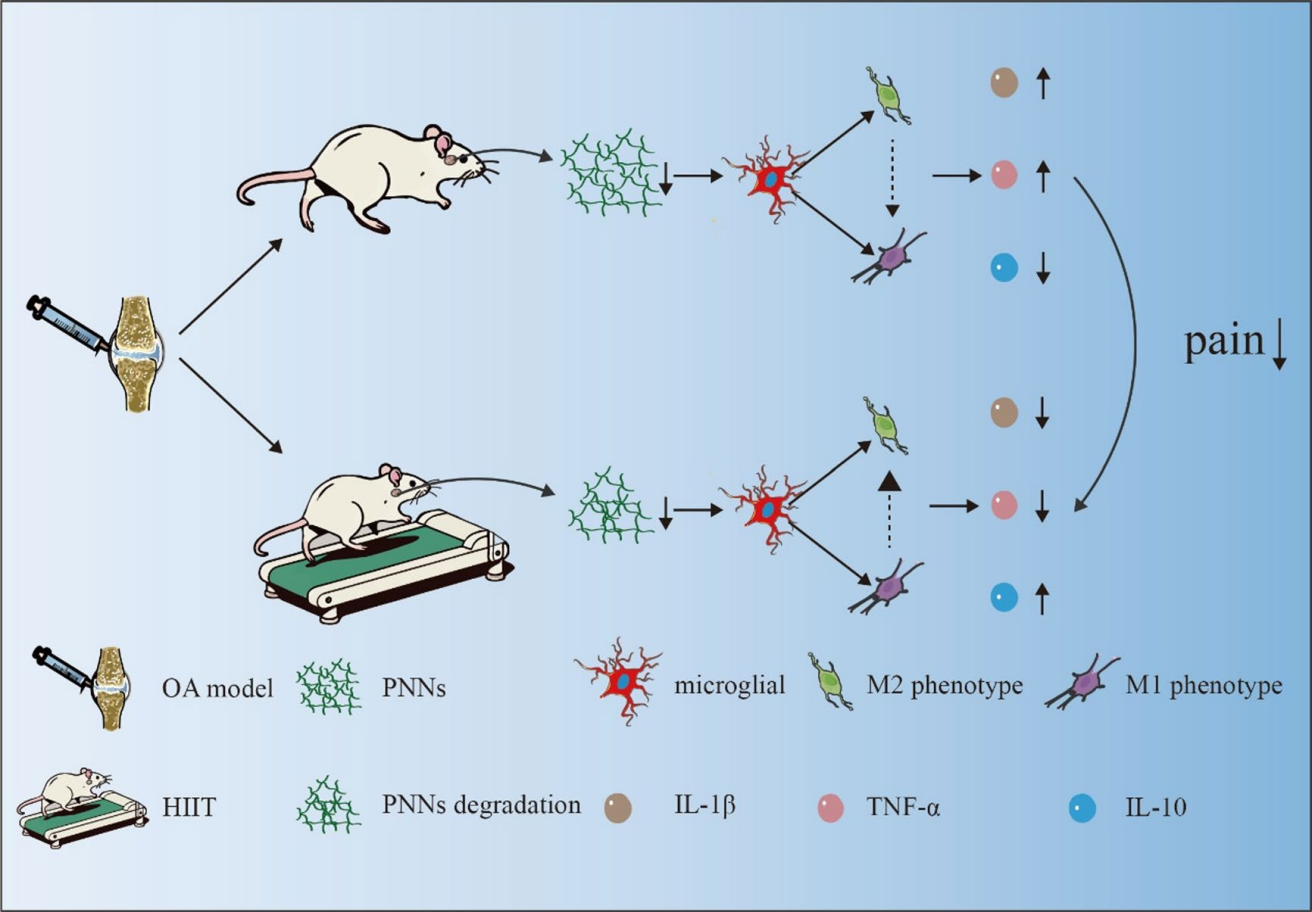
Proposed mechanism of HIIT’s alleviation of OA-related pain via modulation of PNNs and microglial polarization in the mPFC.

Collectively, these findings support a model in which HIIT reduces PNN accumulation in the mPFC, thereby reprogramming microglia from a pro-inflammatory to an anti-inflammatory state. This central neuroimmune reorganization alleviates neuroinflammation and contributes to pain relief and joint protection in OA (Fig. 8).

## Discussion

In this study, we explored the impact of HIIT on knee OA in rats, focusing on its impact on PNNs and microglial polarization in the mPFC. Our findings suggest that HIIT alleviates OA-related pain and inflammation through a novel signaling pathway involving PNNs and microglial polarization, providing new insights into the central regulation of pain in OA.

OA is a prevalent degenerative joint disorder defined by progressive cartilage breakdown, synovial inflammation, and persistent pain^28–30^. The management of pain remains a significant challenge in OA, as both peripheral inflammation and central sensitization contribute to pain perception^31–34^. Recent research has highlighted the role of neuroinflammation, particularly in brain regions such as the mPFC, which is involved in both pain processing and emotional regulation^4,35^. In this study, we demonstrate that HIIT mitigates OA-associated pain by targeting central inflammation within the mPFC and modulating the underlying neuroinflammatory pathways.

One of the key findings of our study is the reduction in PNN accumulation and the shift in microglial polarization from a pro-inflammatory (M1) to an anti-inflammatory (M2) phenotype in the mPFC. PNNs, specialized extracellular matrix structures, are crucial for synaptic stability and plasticity^36^. However, excessive PNN integrity can limit neuronal plasticity and exacerbate central pain sensitization^19,37^. Our results show that OA induces an increase in PNN expression in the mPFC, which is associated with an elevated pro-inflammatory microglial phenotype, as evidenced by increased co-localization of Iba1 and iNOS. HIIT significantly reduced PNN expression and reversed microglial polarization towards the anti-inflammatory M2 phenotype, marked by enhanced Arg1 expression and reduced iNOS expression.

The neuroinflammatory effects of HIIT were further confirmed by the reduction of pro-inflammatory cytokines such as IL-1β and TNF-α, alongside an increase in anti-inflammatory cytokines like IL-10 in both serum and synovial fluid^38,39^. These changes are consistent with the alleviation of the overall inflammatory response in OA rats following HIIT. Importantly, the shift in microglial polarization from M1 to M2 suggests that HIIT not only modulates peripheral inflammation but also exerts central effects by promoting an anti-inflammatory neuroenvironment, which is critical in the management of chronic pain in OA. However, whether HIIT confers sustained long-term benefits beyond the training period remains to be determined in studies with extended follow-up.

Our study also highlights the role of PNNs in modulating microglial activation and neuroinflammation in OA. We demonstrated that degradation of PNNs in the mPFC using ChABC reduced microglial activation and promoted an anti-inflammatory phenotype. Interestingly, no additive effect was observed when ChABC was combined with HIIT, suggesting that PNN degradation may mediate the effects of HIIT on microglial polarization. These findings provide a new perspective on the role of PNNs in the central regulation of inflammation and pain, presenting them as potential therapeutic targets in OA.

In addition to its effects on the mPFC, HIIT also ameliorated cartilage degeneration and reduced knee joint swelling in OA rats. Histological analysis showed that HIIT mitigated cartilage damage, as evidenced by reduced MMP13 expression and increased COL2A1 levels. This finding is consistent with previous studies that suggest exercise can influence both peripheral and central aspects of OA pathology^40^. The reduction in systemic inflammation, as evidenced by decreased levels of IL-1β and TNF-α in serum and synovial fluid, further supports the anti-inflammatory effects of HIIT. Clinically, HIIT-like protocols may be adapted as individualized, low-impact interval exercise with gradual progression, but feasibility and safety in OA patients require further clinical evaluation.

While robust across behavioral, histological, and molecular readouts, the precise upstream signals by which HIIT remodels PNNs–and the generalizability of this mechanism to other nociceptive circuits–remain to be defined. Importantly, our experimental design primarily targeted the prevention of OA pain formation (i.e., interventions were initiated during the development phase), rather than evaluating therapeutic efficacy after pain had been fully established. Therefore, whether HIIT, PNN degradation (ChABC), or microglial inhibition (minocycline) can reverse established OA pain and the associated central neuroinflammatory alterations remains unclear. Future studies should apply these interventions after stable pain behaviors are confirmed and incorporate longitudinal follow-up to determine therapeutic reversibility, durability, and dose–response characteristics. To our knowledge, this is the first evidence that exercise-induced analgesia in OA proceeds via a PNN-driven microglial reprogramming sequence in the mPFC, warranting targeted validation and mechanistic dissection.

## Conclusion

In conclusion, our study demonstrates that HIIT alleviates OA-related pain and inflammation through a novel mechanism involving the regulation of PNNs and microglial polarization in the mPFC. By targeting both central and peripheral inflammation, HIIT represents a promising therapeutic strategy for managing chronic pain in OA. These findings not only enhance our understanding of the effects of exercise on OA but also suggest that modulating CNS inflammation and microglial activation may be an effective approach to improving pain management in OA patients.

## Supplementary Information

Below is the link to the electronic supplementary material.

Supplementary Material 1

## Data Availability

Data will be made available on request. Please contact the corresponding author, Xueping Li, at lixueping6504@163.com for data access requests.
