# Supplementary material for: High-intensity interval training remodels perineuronal nets in the medial prefrontal cortex to drive microglial polarization and alleviate osteoarthritis pain
Source: Sci Rep. 2026 Feb 20;16:9983. doi: 10.1038/s41598-026-40823-w (PMC13022222; doi:10.1038/s41598-026-40823-w)

Supplementary Material

Supplementary Figure 1 (Fig.2 E)

COL2A1


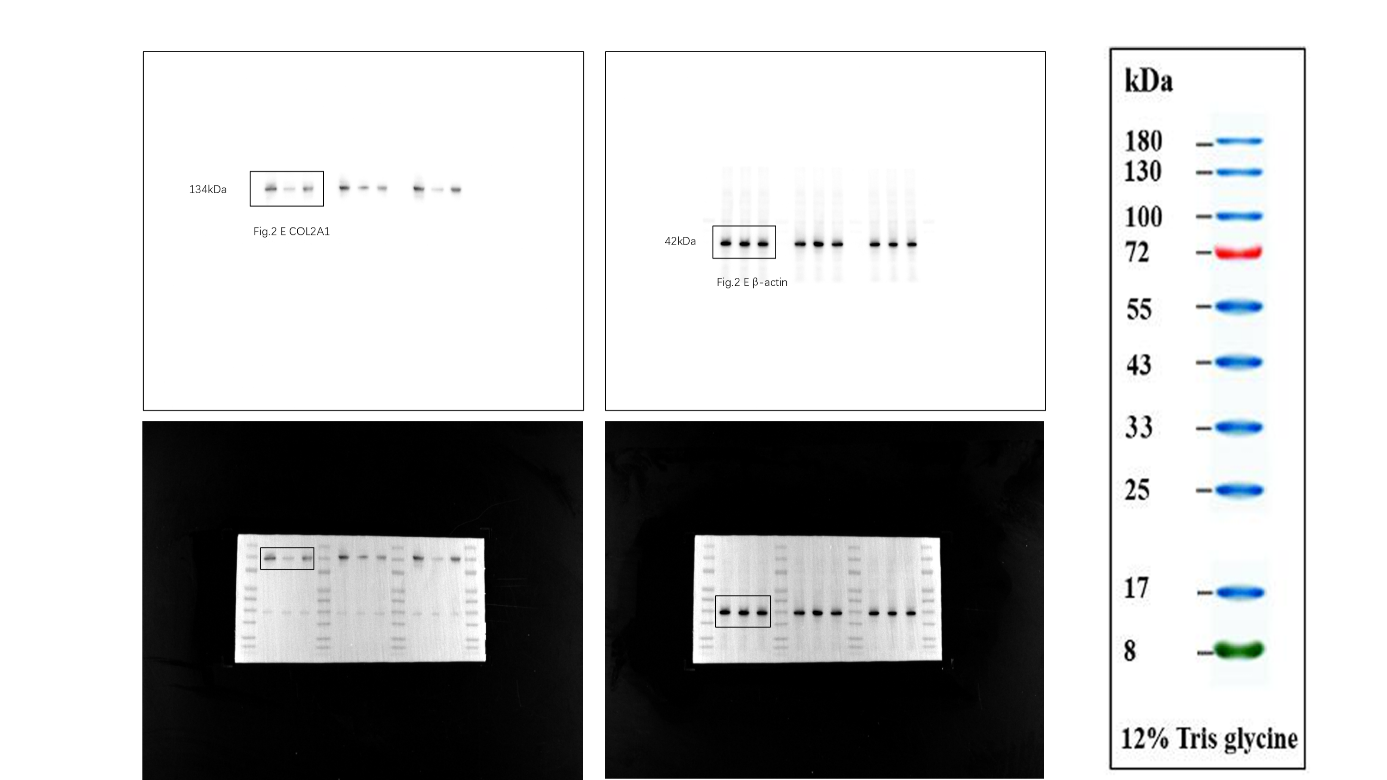


MMP13


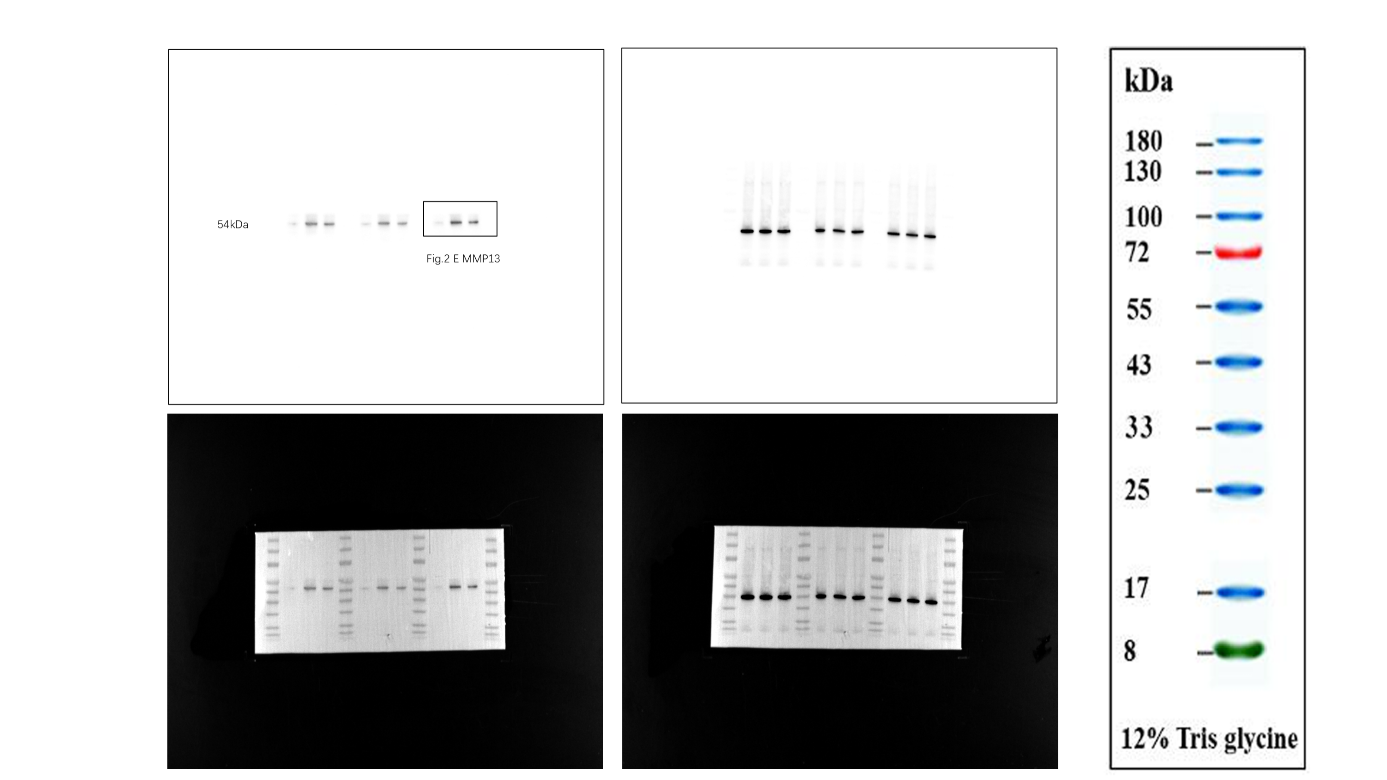


Supplementary Figure 2 (Fig.4 C)

iNOS


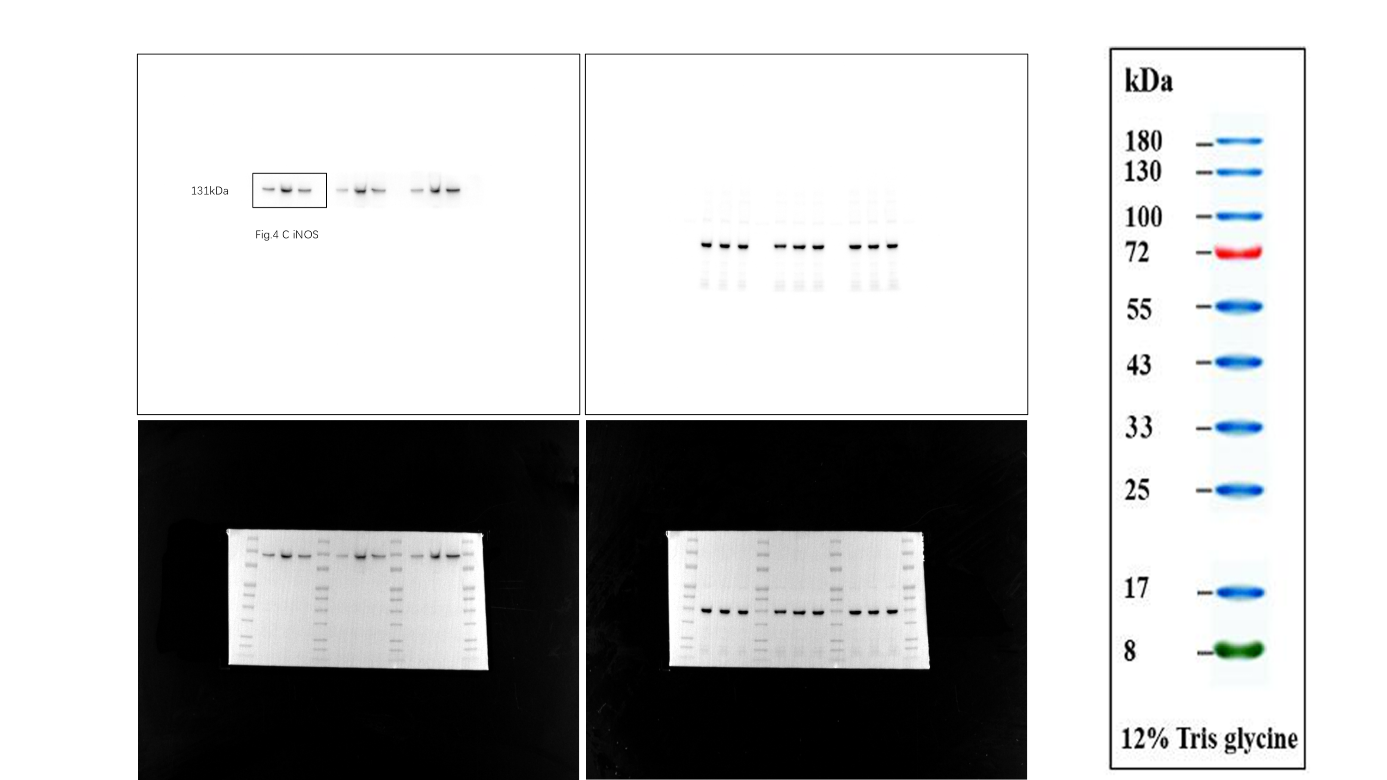


Arg1


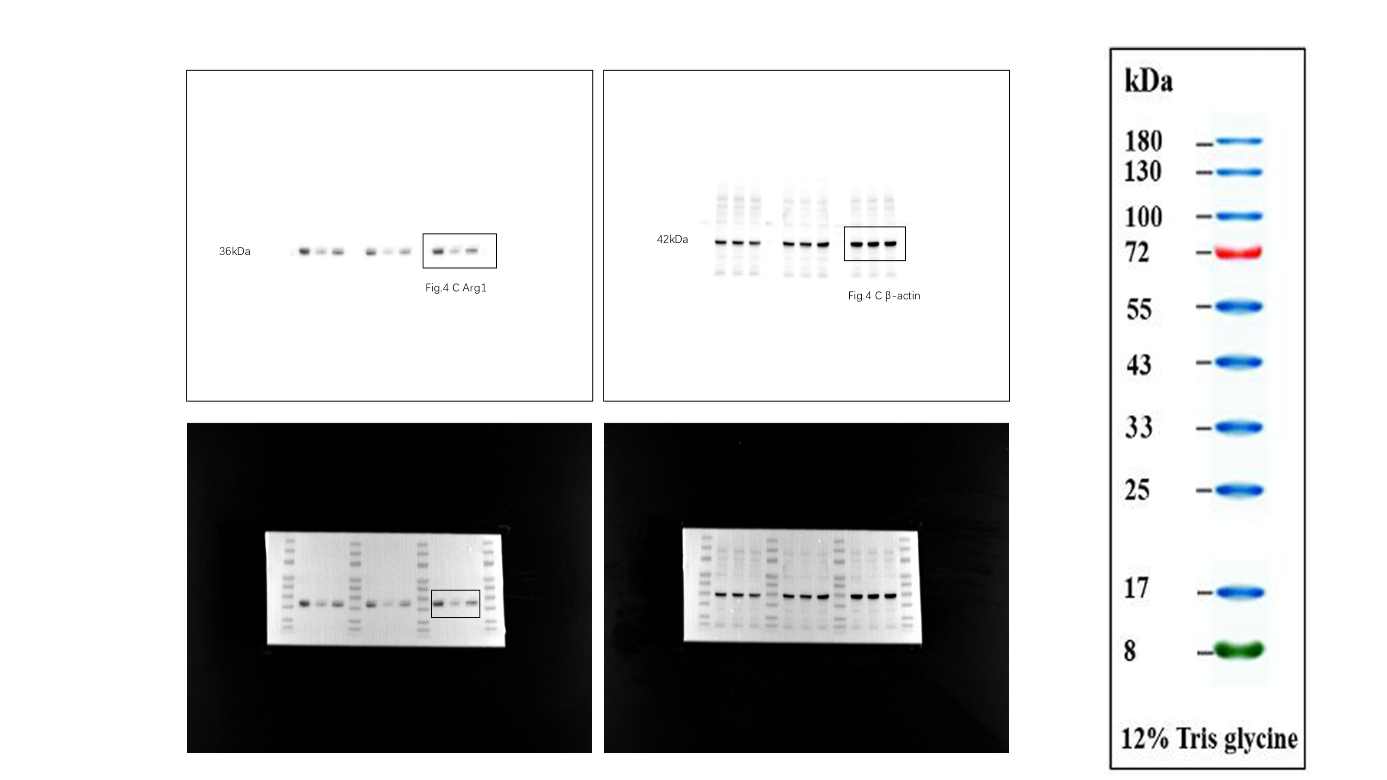


IL-10


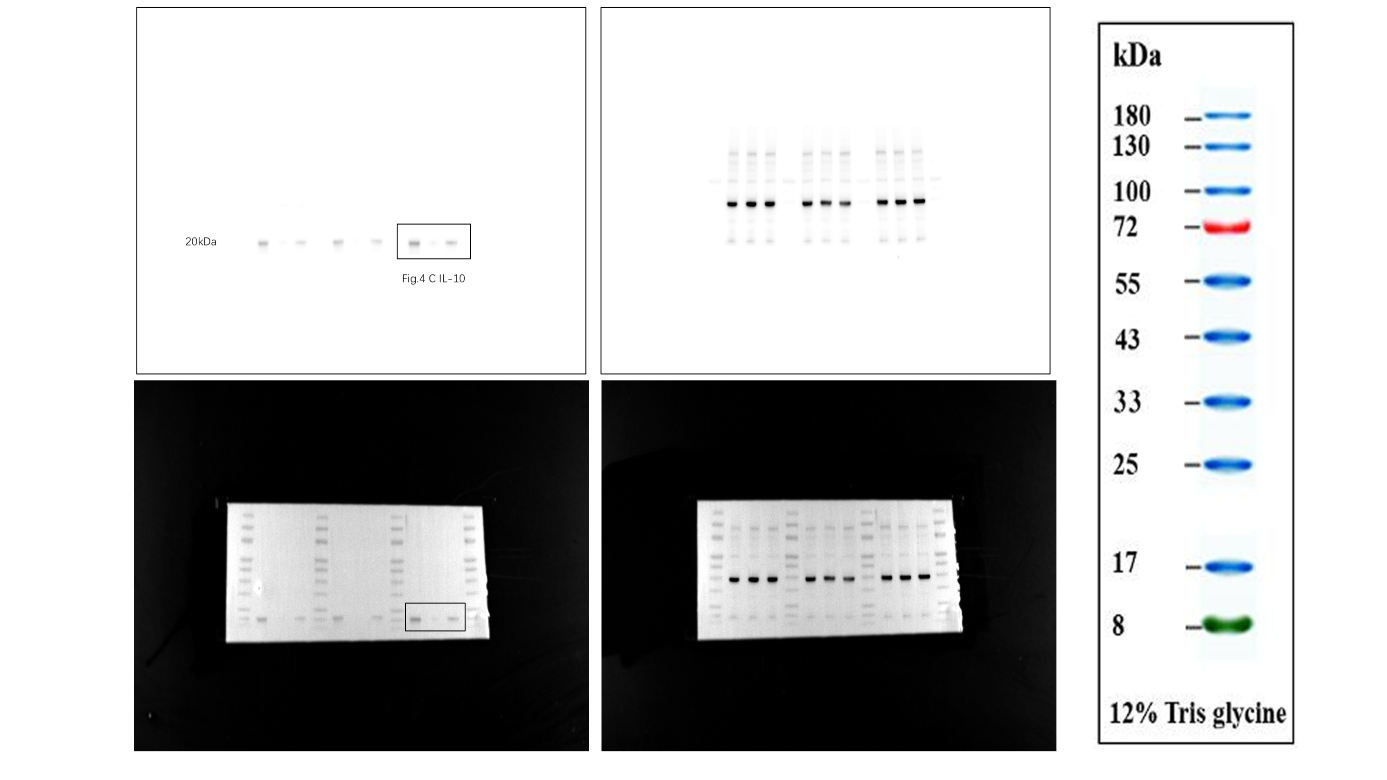


IL-1β


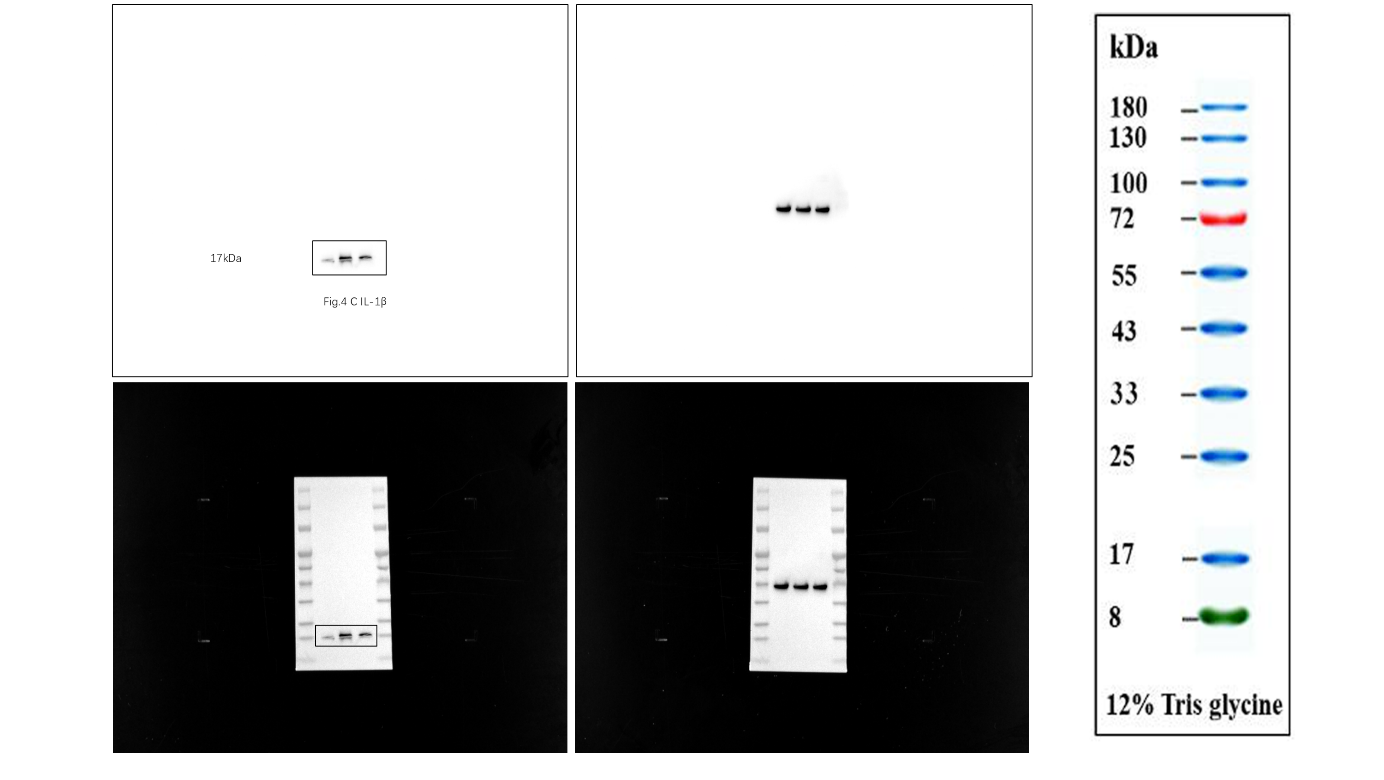


TNF-α


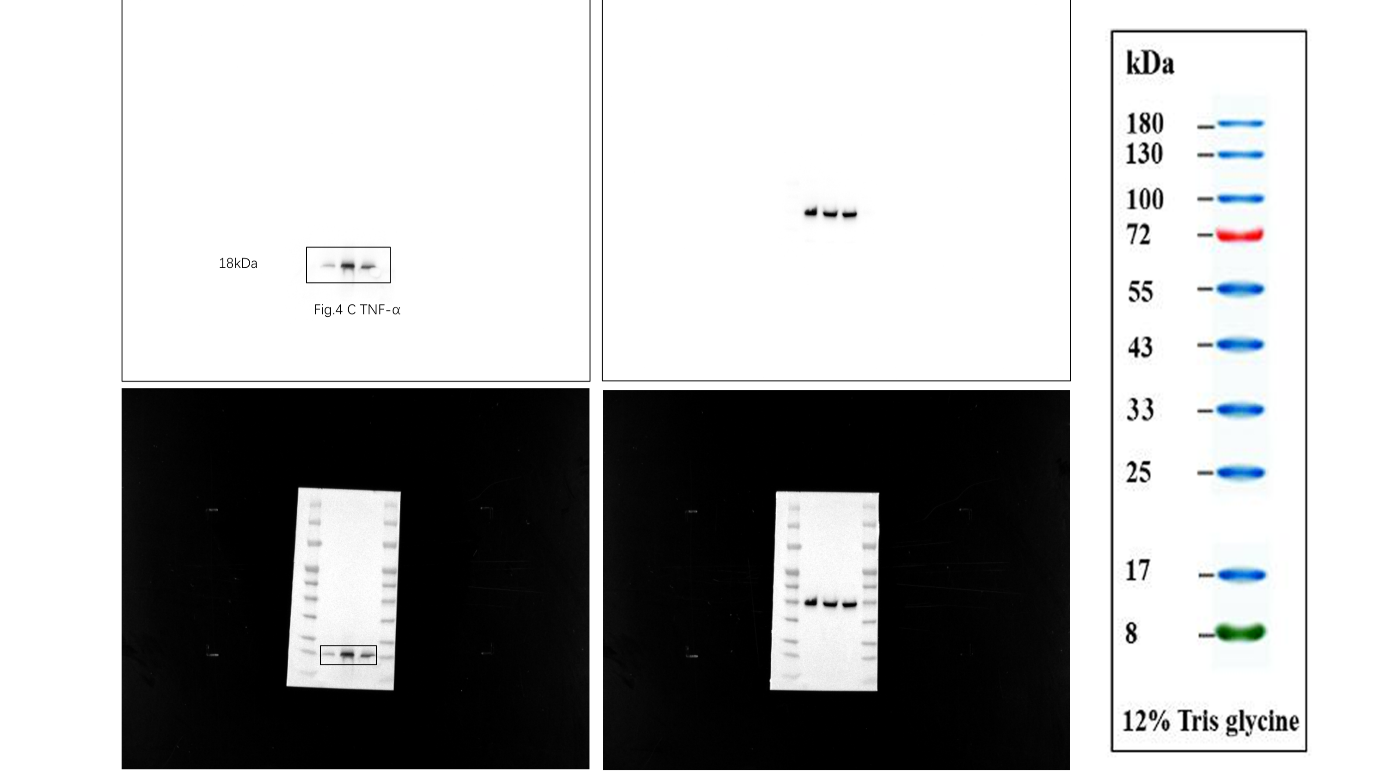


Supplementary Figure 3 (Fig.5 C)

iNOS


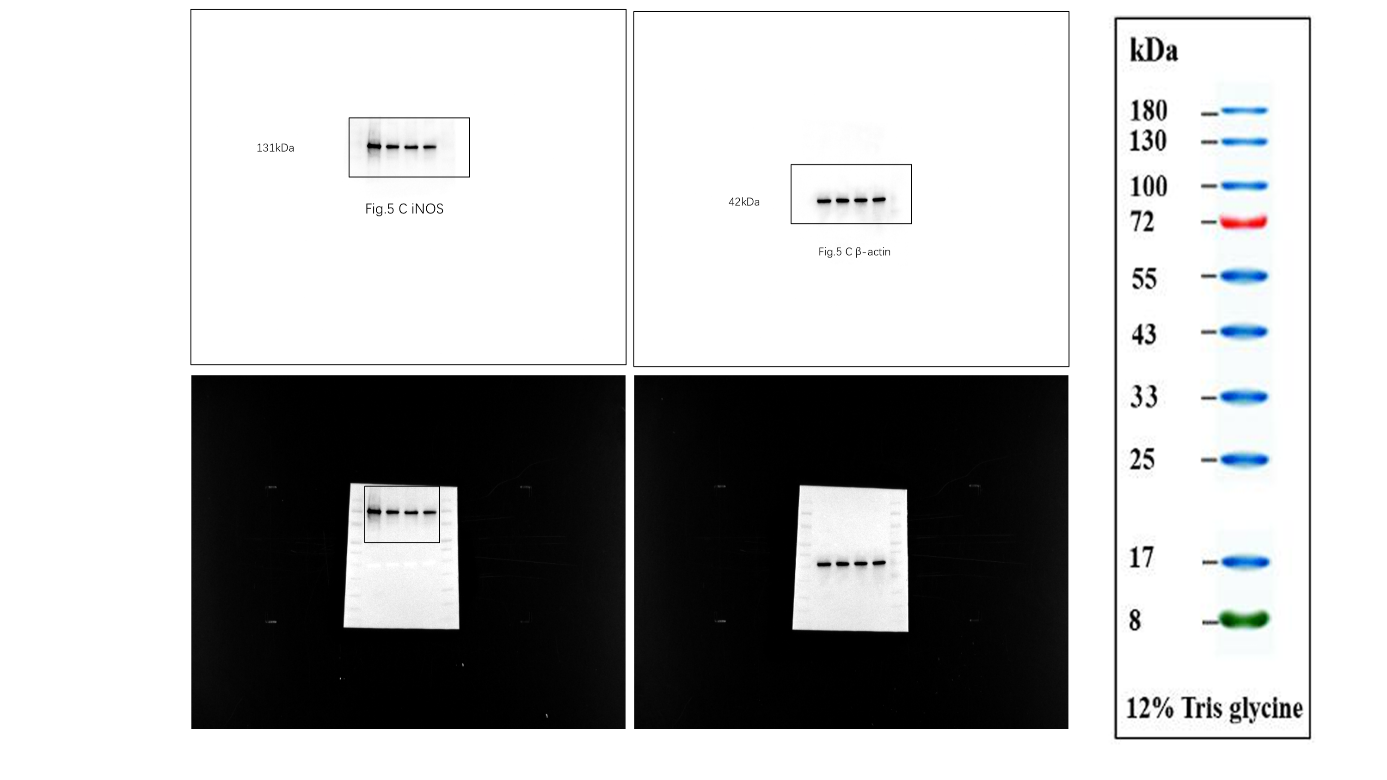


Arg1


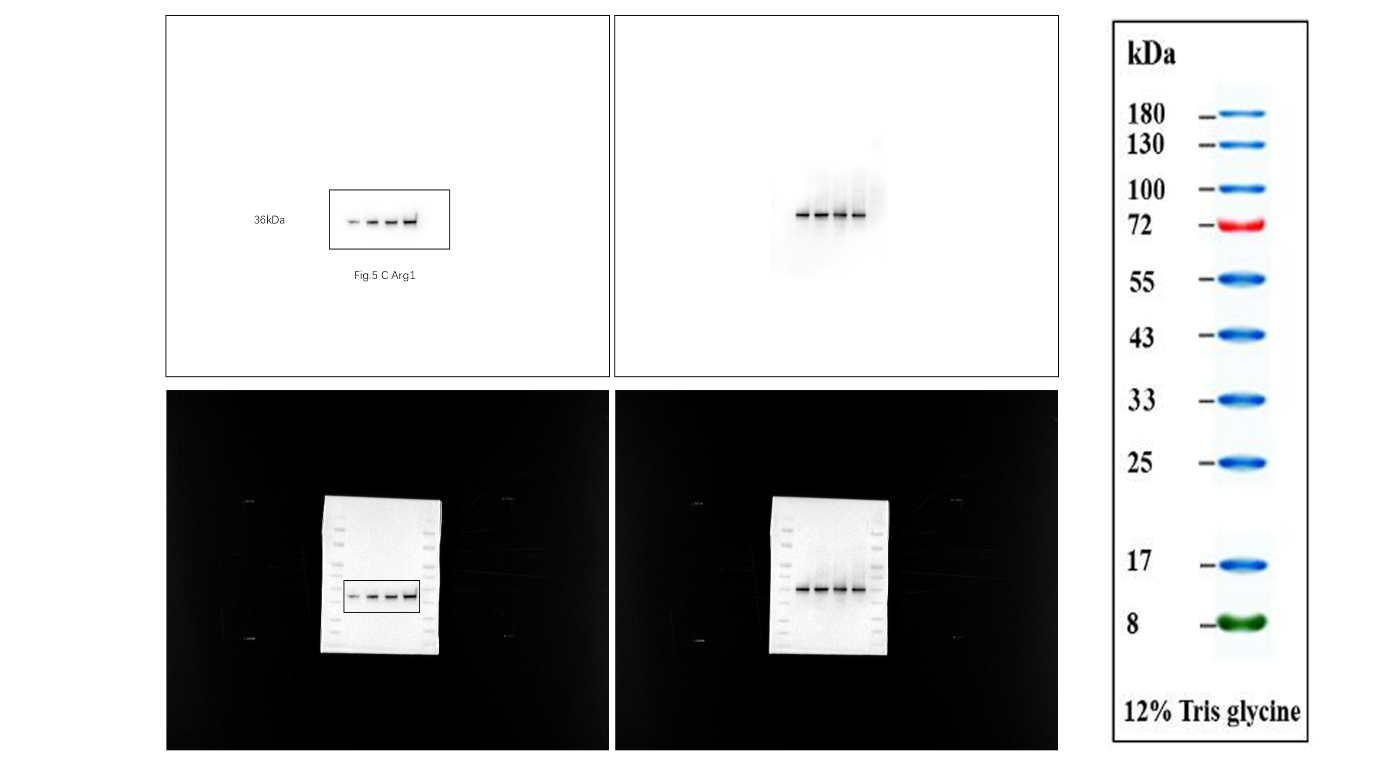


IL-1β


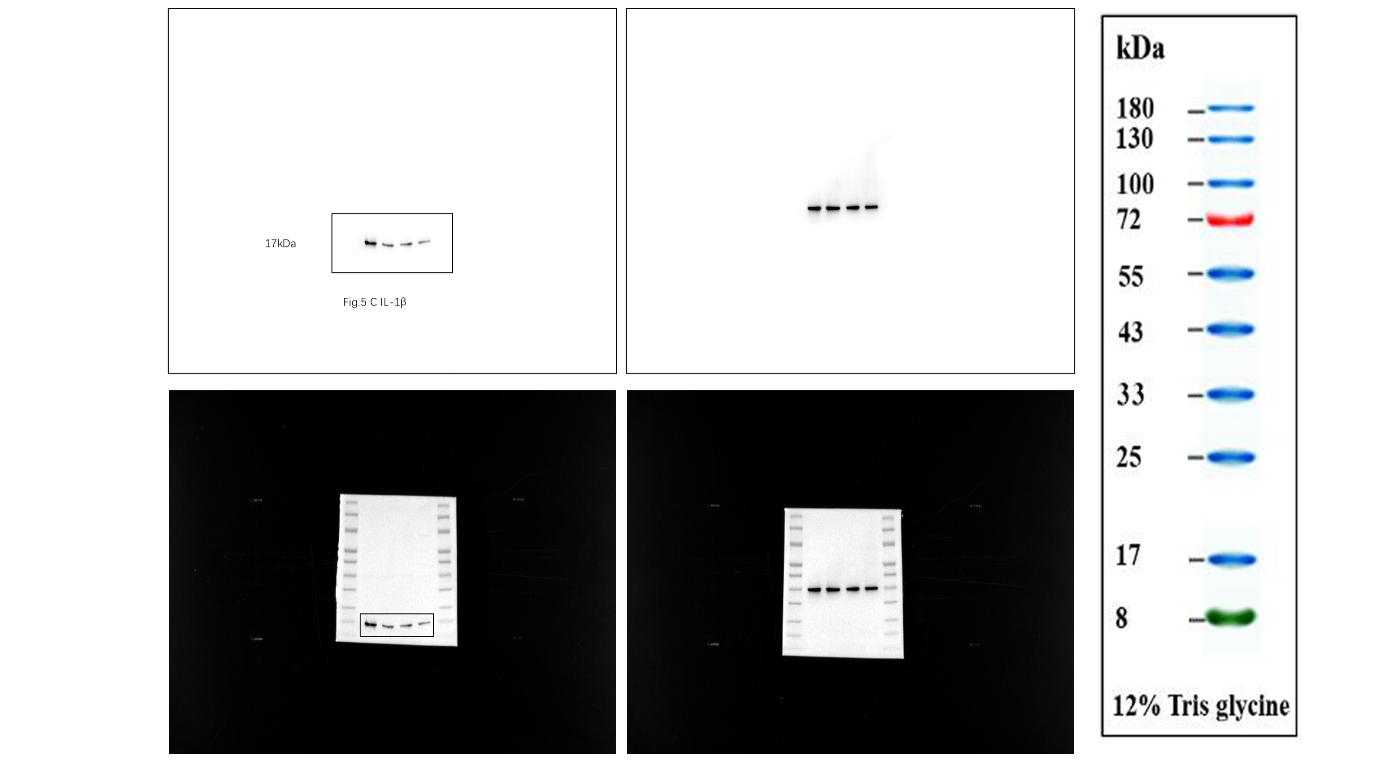


TNF-α


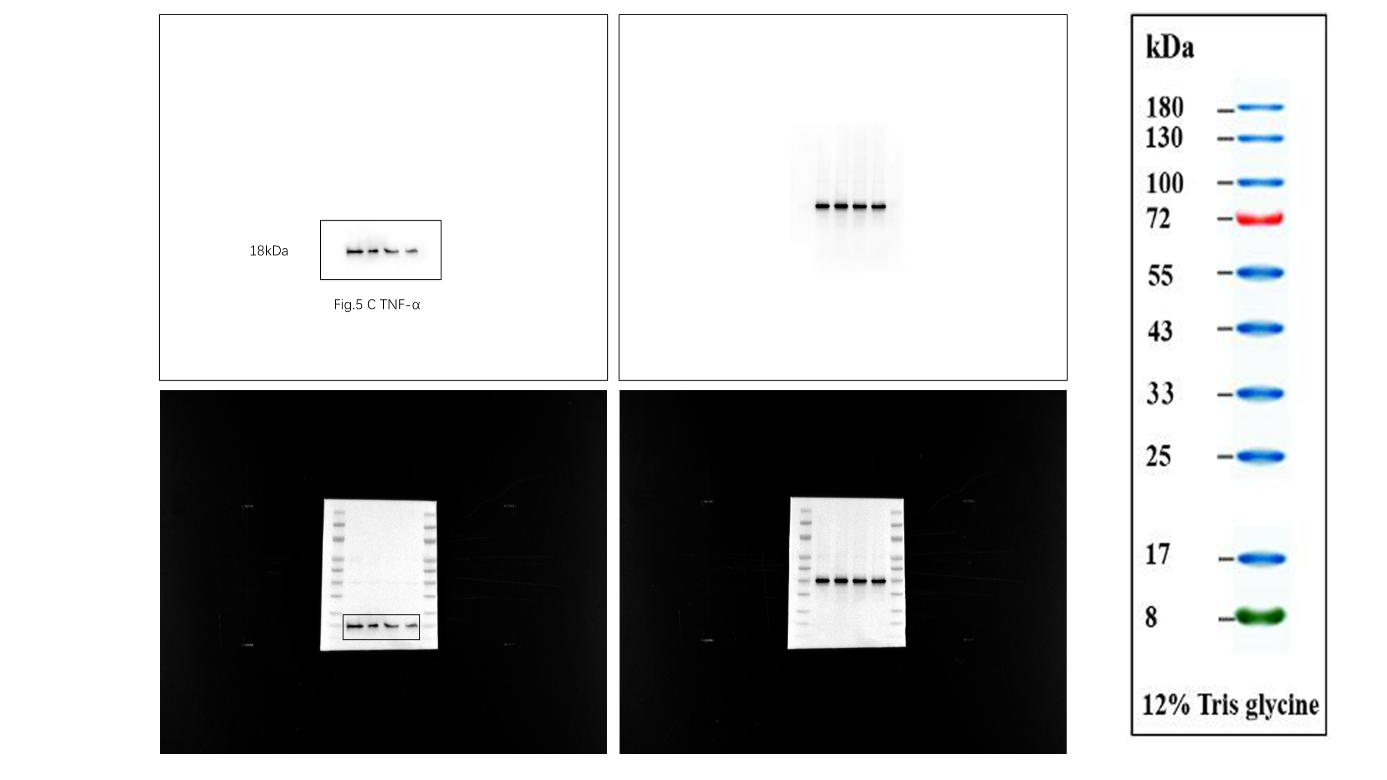


IL-10


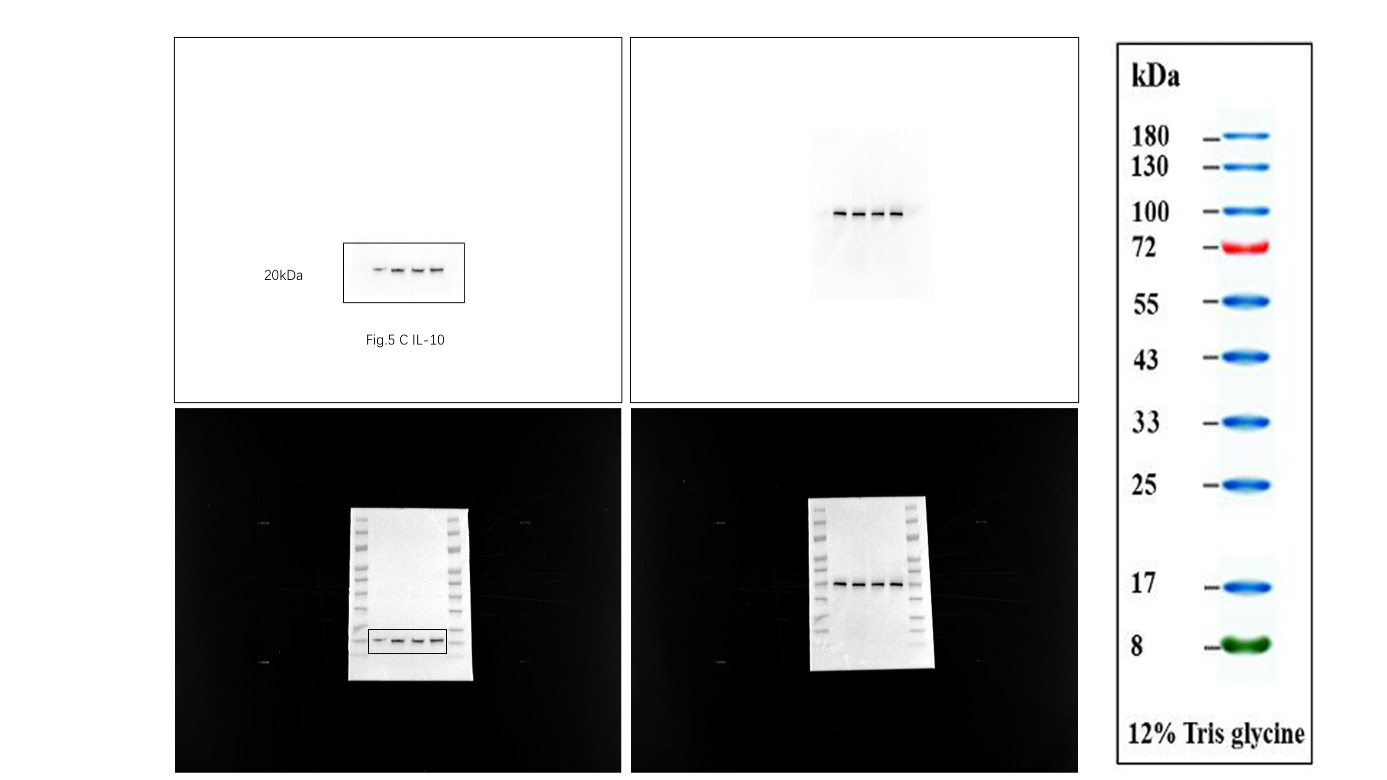


Supplementary Figure 4 (Fig.6 F)

COL2A1


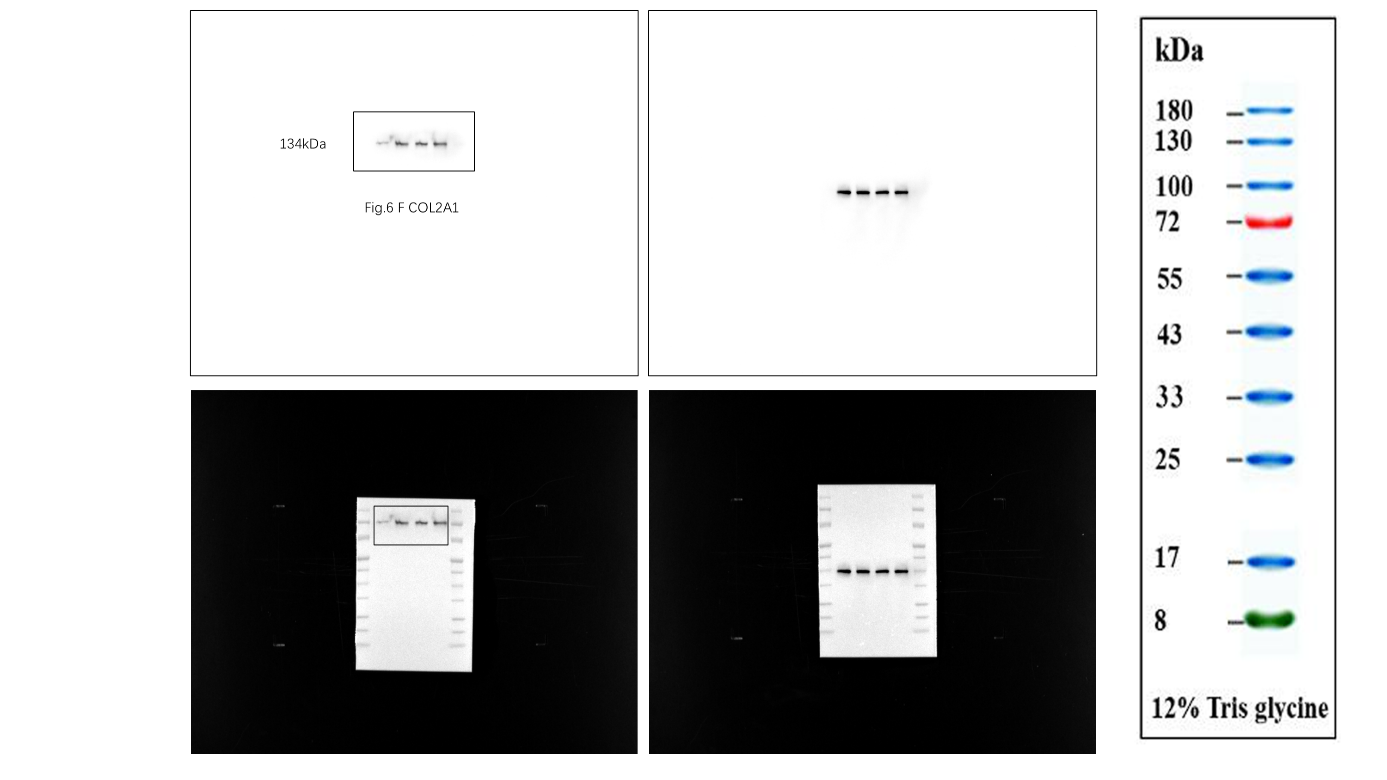


MMP13


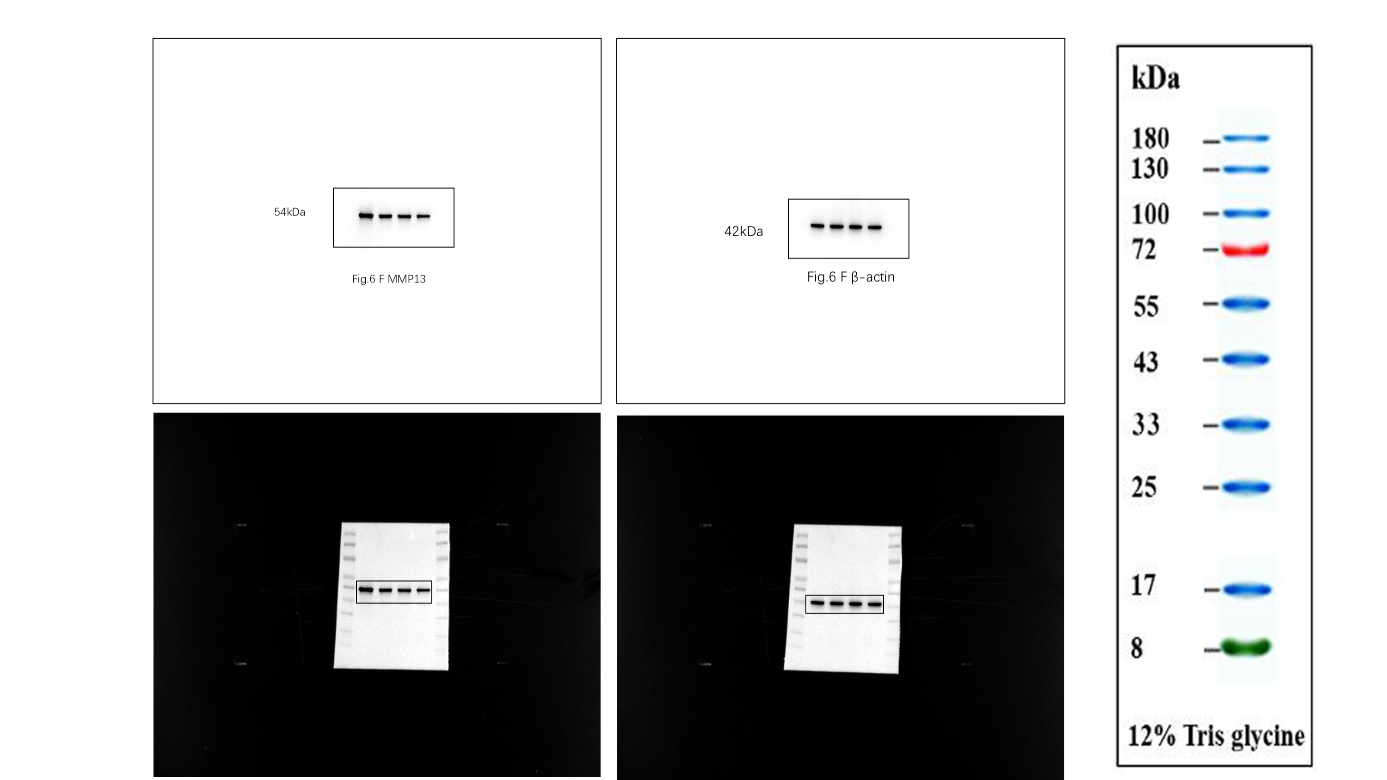

Supplement: Supplementary file 1 — Supplementary Material 1 [file 41598_2026_40823_MOESM1_ESM.docx]
